# Large-scale invasion of unicellular eukaryotic genomes by integrating DNA viruses

**DOI:** 10.1073/pnas.2300465120

**Published:** 2023-04-10

**Authors:** Christopher Bellas, Thomas Hackl, Marie-Sophie Plakolb, Anna Koslová, Matthias G. Fischer, Ruben Sommaruga

**Affiliations:** ^a^Department of Ecology, Universität Innsbruck, 6020 Innsbruck, Austria; ^b^Groningen Institute for Evolutionary Life Sciences, University of Groningen, 9747 AG Groningen, The Netherlands; ^c^Department of Biomolecular Mechanisms, Max Planck Institute for Medical Research, 69120 Heidelberg, Germany

**Keywords:** virus, protist, Polinton, virophage, endogenous

## Abstract

Eukaryotic genomes contain a variety of endogenous viral elements (EVEs), which are mostly derived from RNA and ssDNA viruses that are no longer functional and are considered to be “genomic fossils.” Genomic surveys of EVEs, however, are strongly biased toward animals and plants, whereas protists, which represent the majority of eukaryotic diversity, remain poorly represented. Here, we show that protist genomes harbor tens to thousands of diverse, ~14 to 40 kbp long dsDNA viruses. These EVEs, composed of virophages, Polinton-like viruses, and related entities, have remained hitherto hidden owing to poor sequence conservation between virus groups and their repetitive nature that precluded accurate short-read assembly. We show that long-read sequencing technology is ideal for resolving virus insertions. Many protist EVEs appear intact, and most encode integrases, which suggests that they have actively colonized hosts across the tree of eukaryotes. We also found evidence for gene expression in host transcriptomes and that closely related virophage and Polinton-like virus genomes are abundant in viral metagenomes, indicating that many EVEs are probably functional viruses.

## Significance

Protists are a diverse collection of predominantly unicellular eukaryotic organisms that are not animals, plants, or fungi. They make up most of the eukaryotic tree of life, are major components of nearly all ecosystems and are critical for carbon and nutrient cycling. In this study, we found that large parts of protist genomes are viral in origin and that these viral integrations are comparable in scale to prophage integrations in bacterial genomes. Protist EVEs were distantly related to virophages, a group of viruses which parasitize on larger “giant viruses” that infect and kill their eukaryotic hosts. Many EVEs appear to be functional viruses, which suggests that diverse arrays of these elements may be part of a host antivirus system.

Most known endogenous viruses in eukaryotes belong to retroviruses (1), a group of RNA viruses that require integration into a vertebrate genome to complete their life cycle. Occasionally, they enter host germ line cells and over time, numerous entities can become fixed into the genome (2). In this way, up to 8% of the human genome has been acquired from retroviral integrations (3). In unicellular eukaryotes, there are few reports of such substantial virus integrations; however, a variety of endogenous viruses have been detected (1). The largest endogenous viral elements (EVEs) come from nucleocytoplasmic large DNA viruses (NCLDVs), also known as “giant viruses”, which have been reported as up to 2 Mbp insertions in certain chlorophyte genomes. These large, but incomplete viral fragments contribute to genomic variability between strains (4, 5). Sporadic evidence for NCLDV EVEs has also been detected in various protists (including amoeba and algae) as well as in a cnidarian (6–8), suggesting they may be widespread.

A variety of small (15 to 20 kb) virus-like mobile elements have been repeatedly and independently discovered in certain eukaryotic genomes over the last two decades: Tlr elements in the ciliate *Tetrahymena thermophila* (9); Mavericks in mainly vertebrates and invertebrates (10, 11); and the largely synonymous Polintons and Adintoviruses in animal and a few protist genomes (12, 13). Together, we refer to these entities as “Maverick–Polintons,” which were originally thought to be self-synthesizing transposons due to their lack of detectable viral capsid genes (12). However, the detection of virus hallmark genes, including major capsid proteins (MCPs), minor capsid proteins (mCPs), and ATPases now suggests some, if not all, are endogenous viruses (13–15). A notable exception is the parabasalid *Trichomonas vaginalis*, which contains hundreds of Maverick–Polinton elements that make up approximately one-third of the genome (11), but for which no virus capsid genes were found so far. This has supported the idea that some elements lost their viral genes to fully lead a transposon lifestyle and replicate only within a genome (16).

Polinton-like viruses (PLVs) are a relatively new type of virus that have been found to be highly diverse and relatively abundant in aquatic ecosystems (17, 18). As their name suggests, PLVs are distant relatives of Maverick–Polintons, having a similar genomic complement (17–20). However, multiple groups of PLVs exist, which exhibit little detectable sequence similarity with each other, suggesting that they represent several groups of viruses (17, 18). For most of these groups, their presumably unicellular eukaryotic hosts have remained unknown, although related entities have been described in a handful of protist genomes (13, 17, 18, 20, 21). PLVs also share a similar gene content, albeit without detectable sequence similarity, with viruses of the class *Maveriviricetes*, more commonly described as virophages, which are large-virus-dependent or associated viruses (22). All known virophage isolates require a coinfecting giant virus in a eukaryotic host to carry out their replication in cytoplasmic virion factories (23, 24). While there are approximately five different virophage/giant-virus/host systems in culture (25–29), only two known PLV isolates have been characterized: Tetraselmis striata virus (TsV-N1) with a reported lytic infection of the marine algae *Tetraselmis striata* (30), and Phaeocystis globosa virus virophage (PgVV) (31), which is associated with the marine haptophyte *Phaeocystis globosa*. Recent experimentation has shown that PgVV (Gezel-14T) depends on a coinfecting giant virus for its replication and hence has a virophage-like lifestyle (21). Relatives of TsV-N1 and PgVV-type PLVs have recently been found to occur as integrated viruses in *Tetraselmis* spp. and other chlorophyte genomes (20, 21), confirming that they independently colonize their eukaryotic hosts in a way similar to the mavirus virophage in the marine stramenopile *Cafeteria burkhardae* (32).

While attempting to improve host prediction of unique PLVs (17), we noted that virophage, PLV, and Maverick–Polinton MCP genes frequently appeared in draft protist genome assemblies (GenBank Whole Genome Shotgun – WGS database), but were often absent in the final published genomes (GenBank nr; RefSeq databases). To investigate this, we systematically screened all publicly available draft protist genome assemblies for virophage and Polinton-like virus MCP genes, finding that endogenous viruses were hidden throughout repetitive, difficult-to-assemble regions of unicellular eukaryotic genomes, which are often filtered out as contamination. In some organisms, we detected thousands of integrated viruses, implying these viruses make up a significant and previously unrecognized proportion of protist genomes.

## Results

### Detecting Integrated Viruses.

**Fig. 1.**
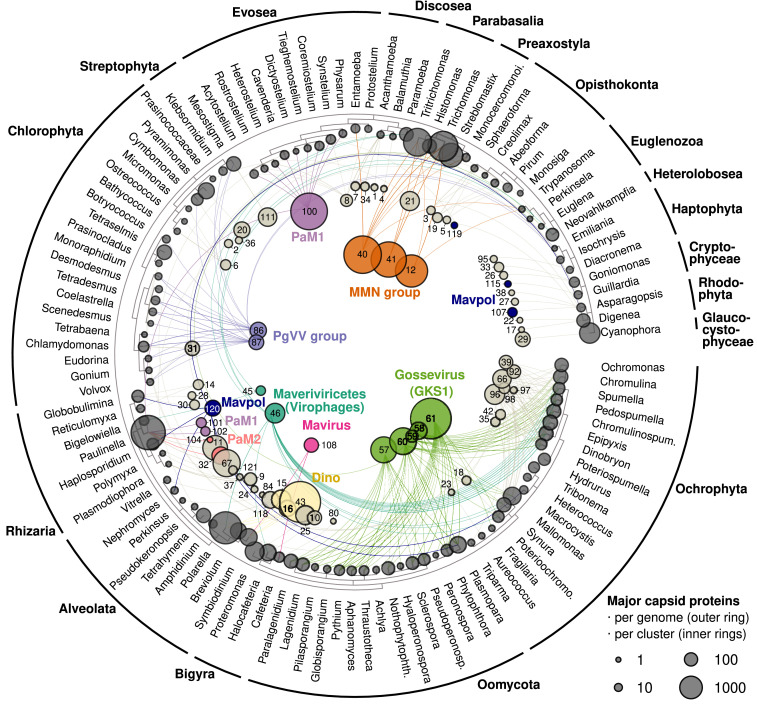
Distribution of endogenous MCP clusters across 462 genomes from 238 protist species. MCP clusters (25% identity across 30% length, 73 clusters) are shown as inner circles scaled in size according to the number of MCPs in each cluster. Numbers refer to the MCP cluster number assigned, with the larger viral groups labeled. MCP clusters are connected to the eukaryotic species (based on NCBI taxonomy) they are found in. Clusters with a more diverse host range are drawn closer to the center of the plot.

To search for virus integrations in unicellular eukaryotic genomes, we downloaded all draft protist genome assemblies from GenBank WGS (1352, December 2021; *SI Appendix*, Table S1) and iteratively searched them for PLV and virophage MCP genes using profile Hidden Markov Models (HMMs) (*Materials and Methods*). Hits were clustered (MMseqs2) (33) and representative sequences were checked using HHpred (https://toolkit.tuebingen.mpg.de/tools/hhpred) to confirm them as double jelly-roll fold MCP genes (*SI Appendix*, Table S2). Where no homology was found, the protein structure was modeled using ColabFold (34) or AlphaFold Colab (35) (https://colab.research.google.com/github/deepmind/alphafold/blob/main/notebooks/AlphaFold.ipynb) and queried against the Protein Data Bank (PDB100) using Foldseek (36) to confirm the gene as a viral MCP gene (*SI Appendix*, Table S3). All draft protist genomes were then searched again against the confirmed virus MCP database using DIAMOND BLASTX (37). In this way, we detected over 35,000 endogenous virus MCP gene hits in 462 protist genomes, which grouped into 73 protein clusters (25% identity over 30% length; MMseqs2) (Fig. 1 and *SI Appendix*, Table S4).

Our analysis identified many highly divergent virus MCP genes in protist genomes, including the missing MCP gene from *T. vaginalis* Maverick–Polintons. The high copy number of Maverick–Polintons in *T. vaginalis*, combined with the lack of viral capsid genes, has remained one of the last major arguments for their existence as transposons (16). We found that one of the conserved hypothetical proteins (DUF4106 family) corresponds to the MCP gene, which is present at 2,224 and 4,841 copies in the two available *T. vaginalis* genomes (WGS accession numbers MJMQ01 and AAHC01). The putative MCP structure prediction contains two eight-stranded, antiparallel β-barrels consistent with other double jelly-roll fold MCP genes (*SI Appendix*, Fig. S1 and Table S3). The existence of a capsid gene in these *T. vaginalis* elements suggests that all Maverick–Polintons are actually endogenous viruses.

The number of viral capsid genes per genome ranged from just a few MCP copies in the smallest free-living eukaryote *Ostreococcus* sp. (Chlorophyta, 13 Mbp genome, 1 to 2 MCPs) to thousands of copies in large genomes such as *Polarella glacialis* (Dinophyceae, ≈3 Gbp genome, 2,535 MCPs) and *Paulinella micropora* (Rhizaria, ≈1 Gbp genome, 3,251 MCPs) (*SI Appendix*, Table S4). The large repetitive nature of dinoflagellate genomes is well known, but our analysis predicts that 1 to 2% of the *P. glacialis* genome may consist of endogenous DNA viruses, assuming all detected MCPs are each part of a 15 to 20 kbp long element. This estimate is even higher for the rhizarian *P. micropora*, where it reaches ≈10% of the genome (*SI Appendix*, Table S4). The most unusual genome was that of *T. vaginalis* (Parabasalia, 160 Mbp genome, 4,841 MCPs), which contained by far the greatest density of MCP genes (27 per Mbp of genomic data). Based on DNA polymerase genes, it has previously been estimated that one-third of its genome consists of Maverick–Polinton elements (11), and our estimates based on MCP gene copies suggest a similar range of 27 to 54%. Similarly high proportions were also found in *Tritrichomonas foetus* (34%). Of particular note were chrysophytes (33 to 104 MCP copies per genome), which contained up to 10 different types of MCP genes (25% identity clusters) per genome, indicating viruses from several diverse groups are capable of integrating into these organisms. Oomycetes, which have been subject to considerable sequencing effort (245 genomes in the dataset), also contained up to 299 MCP copies in a single genome. MCP genes from oomycetes were restricted to five protein clusters (Fig. 1), previously defined as the distinct Gossevirus (GKS1) group of PLVs (17). In the chlorophytes, the dominant type of MCP gene (protein cluster 86 and 87; *SI Appendix*, Table S4) belonged to members of the PgVV group of PLVs (18), suggesting that this group is mainly algal associated. PgVV-type MCP genes have recently been detected in several chlorophyte genomes (21) and our methods confirmed these findings (*SI Appendix*, Table S4). Surprisingly, virophages (defined as members of the *Maveriviricetes* class) were also abundant as endogenous viruses, in particular in *Paramoeba pemaquidensis* (*Neoparamoeba pemaquidensis*, Amoeba, 64 virophage MCPs), *Neovahlkampfia damariscottae* (Discoba, 21 MCPs), *Halocafeteria seosinensis* (Bigyra, 74 MCPs), and in multiple chrysophyte genomes (0 to 37 MCPs per genome). The dinoflagellates *Symbiodinium microadriaticum* and *Symbiodinium* sp. KB8 also contained mavirus-like virophage MCP genes (2 and 4 MCPs, respectively), in addition to those previously described in *Cafeteria burkhardae* (38), which were also detected by our approach.

### MCP Genes Represent EVEs.

**Fig. 2.**
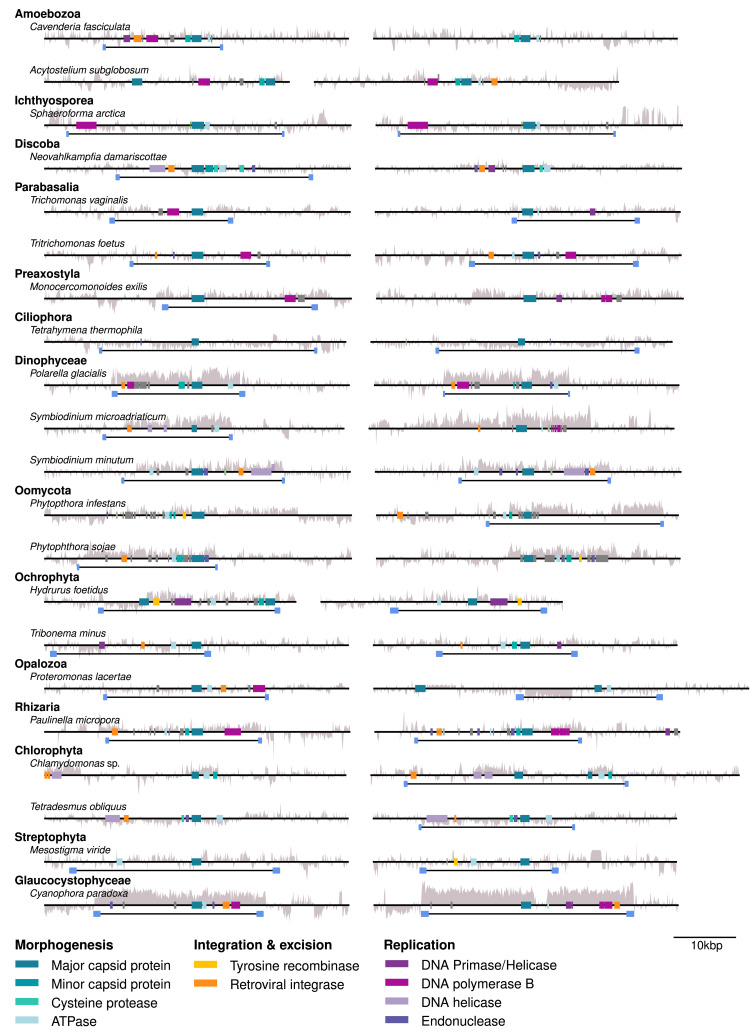
MCP genes are part of complete EVEs. Representative examples of protist-encoded MCP genes (dark teal) are shown in their genomic neighborhood from various eukaryotic groups. EVEs were annotated against a database of known Polinton-like and virophage genes using gggenomes (https://github.com/thackl/gggenomes). TIRs (blue boxes linked by black lines) often border the EVE insertions. The gray graph denotes deviation in GC content from the genomic mean.

To confirm whether MCP genes represent full-length viruses in protist genomes, the MCP gene and surrounding genomic region were extracted from each contig; annotated against a database of PLV, virophage, and Maverick–Polinton genes; and scanned for terminal inverted repeats (TIRs) (*Materials and Methods*). Where the contigs were long enough, our results show that MCP genes were usually present in conjunction with several other PLV/virophage genes, often including a DNA polymerase (pPolB) or DNA primase–helicase (Pri–Hel), and a gene responsible for integration, either a tyrosine recombinase (YR) or a retroelement rve-type integrase. The insertion was generally 15 to 30 kbp in length and sometimes exhibited a notable difference in GC content compared to adjacent host sequences (Fig. 2 and Dataset S1). Approximately 47% (3,487/7,382) of high-quality EVE locations had TIRs (39), which are hallmarks of an inserted element (high quality: contig > 60 kb with MCP > 20 kb from either end, and at least three viral-like genes). These findings allowed us to conclude that the presence of a viral MCP gene is usually also indicative of the presence of a full-length EVE.

### Are Virus Insertions Degraded Elements?

Detecting so many integrated viruses in unicellular eukaryotes raises the question of whether they are intact or degraded (pseudogenized) elements. Many virus genes (including MCP genes) from our automated (Fig. 2) and selected manual annotations (*SI Appendix*, Fig. S2) contained multiple stop codons that initially suggested the latter. However, most protist genome assemblies to date still rely on the assembly of Illumina short reads, making it challenging to resolve closely related and high copy number elements that can be up to 40 kbp long. Indeed, 17% of all hits were found on short contigs, which encoded the MCP gene alone (<1,600 bp) and nearly half of all MCP hits were found on genome fragments that could be considered no larger than the virus genome itself (<25,000 bp). This suggests that the assembly of EVE-containing regions is particularly challenging and causes the viral genomes to assemble as separate contigs. To resolve integrated viruses from short gene fragments, we selected two ochrophyte genomes where we found multiple, diverse MCP genes on short contigs deposited in GenBank WGS, and for which additional long-read (PACBIO) sequencing data were publicly available. We screened PACBIO long reads from *Pedospumella encystans* strain JBM/S11 (SRR9203571) and *Synura* sp. LO234KE (SRR9203568) for virus capsids and error-corrected several matching ~10 kbp long reads (*Materials and Methods*). This produced long contigs with multiple complete viral genes, showing that lone MCP genes detected in GenBank WGS are poorly assembled fragments of larger viral insertions (*SI Appendix*, Fig. S2). To further assess the observation of fragmented EVE genes, we downloaded the longest EVE assembled from the chrysophyte *Hydrurus foetidus* (GenBank UYFQ010000146; 22 Kbp), which appeared degraded due to numerous premature stop codons. After error-correcting the assembly by mapping with Illumina data using strict criteria (*Materials and Methods*), the initially observed stop codons were no longer present, which also revealed a Ty3/Gypsy retrotransposon embedded into the PLV genome. This implies many stop codons are assembly artifacts, similar to those found in endogenous mavirus-like elements (38). Hence, MCP genes that initially appeared fragmented or degraded are likely to represent larger complete EVEs with intact viral genes. However, error-correcting long reads in this way often failed as Illumina reads from multiple, near-identical genes tend to aggregate onto these endogenous virus regions, creating a false consensus contig with much greater coverage than the host genomic average. The erroneous consensus sequence tends to contain single-nucleotide variations and frameshifts, which cause genes to look artificially fragmented.

**Fig. 3.**
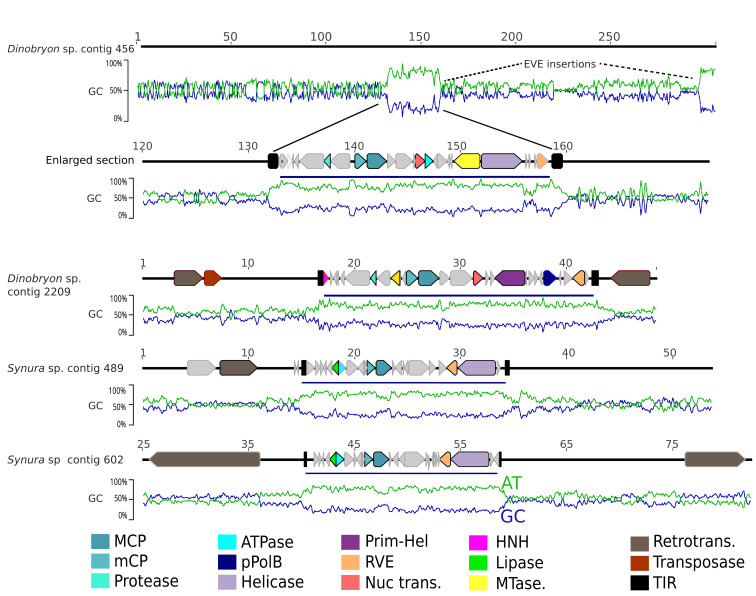
Protist genome assemblies from long-read data resolve full-length EVE insertions. Selected contigs from a partial assembly of Oxford Nanopore reads from *Synura* sp. LO234KE and *Dinobryon* sp. LO226KS showing virus insertion regions flanked by TIRs. Integrated viral regions often exhibit a notable difference in GC content from the host genome. *Synura* sp. contains Polinton-like EVEs, whereas *Dinobryon* sp. EVEs belong to the virophage group. Contig 456 features two insertions at positions 150 and 300 kbp, the latter being only partially assembled. Retrotransposons (Ty3/Gypsy; brown annotation) are often observed near virus insertions. Graphs below contigs show GC (blue) and AT (green) percentages. Numbers denote contig position (kbp). MCP – major capsid protein, mCP – minor capsid protein, Protease – cysteine protease, Helicase – DNA helicase, pPolB – DNA polymerase type B, Pri–Hel – Primase–Helicase, RVE – retroelement type integrase, Nuc trans. – nucleotidyl transferase, MTase – DNA methyltransferase, Retrotrans. – retrotransposon, TIR – terminal inverted repeat, HNH – homing endonuclease.

To further confirm whether MCPs detected on short fragments represent full-length PLVs, we resequenced two ochrophyte genomes using Oxford Nanopore long-read technology (*Materials and Methods*). These genomes were selected based on numerous MCP gene hits to viruses detected in an alpine lake (17). *Dinobryon* sp. (LO226KS) (chrysophyte) and *Synura* sp. LO234KE (synurid) partial assemblies were screened for MCP genes using DIAMOND BLASTX (37) (*Materials and Methods*), resulting in 58 and 61 MCP hits per genome, respectively. This compares well to 46 (*Synura* sp.) and 75 (*Dinobryon* sp.) MCPs detected in our original searches of publicly available assemblies generated from short-read data (*SI Appendix*, Table S4). Crucially, however, our polished long-read assemblies (*Materials and Methods*) resulted in longer contigs (n50 values of 44 kbp and 79 kbp, respectively, compared to 2 kbp and 2.5 kbp in GenBank WGS), often containing complete virus genomes integrated into larger host contigs (39). Where the contigs were long enough, a drop in GC content for 20 to 30 kbp often denoted the boundary of the insertion sequence (Fig. 3).

### EVE Diversity within a Single Protist Genome.

We here show that protist genomes can contain thousands of virus MCP genes, many of which probably represent full-length endogenous viruses, but tend to assemble as separate contigs from short-read data. This raises the question as to whether EVEs are high copy number identical elements or a collection of distinct, but closely related elements within a genome. The finding of erroneous stop codons leading to gene fragmentation during assembly of eukaryotic EVEs (*SI Appendix*, Fig. S2) suggests that these elements are not identical within a genome. We chose *P. micropora* strain KR01 (GenBank accession WBZZ01) to examine the diversity of MCP genes within a single organism, as we detected thousands of diverse sequences within this genome (*SI Appendix*, Tables S4 and S5). The genome is well assembled into long contigs (n50 = 143 kbp) and thousands of Maverick–Polinton-like DNA polymerase genes have been previously detected in this genome (40, 41). Using our DIAMOND BLASTX hits, we extracted 2,678 MCP nucleotide sequences longer than 900 bp for analysis (out of 3,251 hits in total), which spanned at least six MCP groups (*SI Appendix*, Table S4), and found that only 49 were identical copies (MMseqs clustering, 100% identity over 30% length) (*Materials and Methods*). Even after clustering sequences at 98% identity, which allowed for any sequencing and assembly errors (*SI Appendix*, Fig. S2), 1,792 unique clusters were generated, implying hundreds of variants exist within a single genome. Indeed, genome-wide MCP diversity was too large to produce a meaningful MCP alignment at the nucleotide level, and premature stop codons precluded full-length amino acid alignments. Hence, we clustered nucleotide sequences at 50% identity (across 30% length) before each cluster was aligned separately. MCP genes formed five main clusters with this threshold, each containing a wide spectrum of diversity, with often tens to hundreds of closely related, but rarely identical, genes coexisting. Each cluster is displayed separately in *SI Appendix*, Figs. S3–S7 to highlight both the large macro- and micro-diversities in a single gene within one organism, which explains the difficulty of accurately assembling EVEs from short-read sequence data.

### Diversity Between Genomes.

To determine whether certain EVEs are associated with specific eukaryotic lineages, or whether they show evidence of mobility between diverse hosts, we ran a network-based analysis of sequence similarity using all endogenous MCP genes (*Materials and Methods*). Such an approach allows for the comparison of thousands of highly diverse genes where multiple sequence alignments are not feasible. In this analysis, we also included MCPs from the originally described Maverick–Polintons (14), Adintoviruses (13), and Maverick–Polintons detected in vertebrate genomes (15) along with MCPs from all metagenomically detected PLVs (17) before performing BLAST-based sequence comparisons (1e-4 cutoff; *Materials and Methods*).

**Fig. 4.**
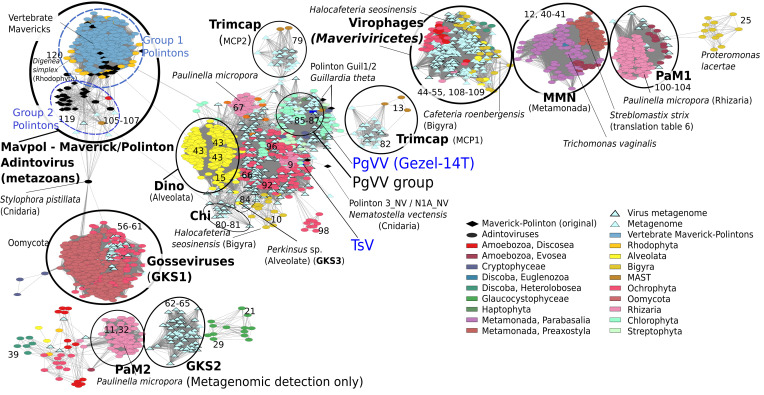
Network analysis showing the relationships between the major capsid proteins from Maverick–Polintons, PLVs, and virophages in eukaryotic genomes. All MCP genes were retrieved from publicly available protist genomes, published works, and environmental metagenomes. To reduce complexity, protist-encoded MCP genes were clustered at 90% nucleotide identity for each individual host genome. Selected species important to the narrative are labeled. Colors represent the genome from which the MCP was retrieved, labeled circles represent the wider virus group to which they belong, designated by MCP type. Light blue diamonds show metagenomically detected MCP genes (17) clustered at 70% identity to reduce numbers, a black border highlights those detected in virus only metagenomes. Initially reported Polinton (14) and Adintovirus (13) MCP genes are shown in black, and vertebrate Mavericks (15) are shown in dark blue (all clustered at 90% identity). Lines (edges) represent a BLASTP alignment at an expectation threshold of <1 × 10^−4^. Blue abbreviations denote isolated viruses: TSV, Tetraselmis striata virus; PgVV, Phaeocystis globosa virus virophage (Gezel-14T). Bold names denote virus groups, with GKS 1-3 and Trimcap referring to groups described by Bellas and Sommaruga (17). MMN, PaM1-2, and Dino are groups of MCP genes defined in this study. Numbers denote major MCP protein clusters as shown in Fig. 1 and in detail in *SI Appendix*, Table S4.

From this analysis (Fig. 4), MCP genes formed multiple, highly diverse groups, suggesting that they represent a broad collection of viruses. *P. micropora* MCPs fell into at least three of these major superclusters, confirming the presence of diverse viral elements within these genomes. Almost all of the original Maverick–Polintons, those from vertebrate genomes (14, 15) and all known Adintoviruses (13), fell into a large supercluster, which could be divided into previously described Group I and Group II Polintons (16). Exceptions were MCP genes from Cnidaria and *Guillardia theta* (cryptophyte), which fell outside this main group. Hence, the original Maverick–Polintons appear to be a distinct group of viruses which, aside from red algal and one Discosea MCP gene, all came from metazoans. Cnidarian MCP genes were the only metazoans to fall outside this main Maverick–Polinton cluster. All virophage-like (*Maveriviricetes*) MCP genes formed a single cluster that had no detectable similarity to the other groups using these thresholds (BLASTP cutoff 1e-4), serving to highlight the large divergence of MCP genes within these elements. The previously described Gossevirus GKS1 group of PLVs, first detected in an alpine lake, was shown to be a distinct virus group by MCP clustering. Oomycetes almost always contained EVEs possessing Gossevirus group MCP genes (MCP clusters 57 to 61), with MCP clusters 58 to 60 also found in the chrysophytes (both are Stramenopiles). Most dinoflagellate EVE MCP genes formed a separate cluster (Dino group), which was distantly related to MCPs found in diverse hosts, including chrysophytes, chlorophytes, streptophytes, haptophytes, and the viral isolate PgVV that coreplicates with a giant DNA virus (21). The Metamonada group was seeded by the MCP we detected in *T. vaginalis*. This group was composed of MCP variants from five species of parabasalids, plus MCPs from *Streblomastix strix,* a termite gut symbiote noted for using a nonstandard genetic code with TAA and TAG codons encoding the amino acid glutamine (42). EVEs in *S. strix* were also found to use the same translation table, indicating a potential long-term association between virus and host in this group. Interestingly, however, several MCPs found in Evosea (Amoebozoa) also clustered within this main group. Indeed, throughout the network, MCP clusters often included EVEs from multiple eukaryotic lineages, indicating that horizontal transfer of these EVEs must have occurred at some point in their evolutionary history.

Metagenomically detected MCP genes from filtered aquatic viromes (17) clustered into several major groups containing EVE-derived MCPs. This includes members from the Gossevirus GKS1 group, GKS3 group (found in Alveolata), Trimcap group (found in unclassified Stramenopiles), PaM1 group (Rhizaria, Amoebozoa), PgVV group (Chlorophyta, Streptophyta), and the central TsV containing cluster (Chlorophyta and Ochrophyta). The similarity between endogenous and virome-derived MCP genes implies that at least some EVEs are capable of virion formation and are hence functional viruses.

### Evidence for Expression of MCP Genes.

To detect evidence for EVE gene expression, we queried protist transcriptomes from the GenBank Transcriptome Shotgun Assembly (TSA) database (773 transcriptomes; December 2021) against our database of MCP genes. We note that protists in GenBank TSA only partially overlapped with those present in GenBank WGS. However, representatives from most major groups were available. We also note that an organism in culture under constant conditions may not be conducive to EVE expression, particularly if these elements have a virophage lifestyle and require challenge by a giant virus to activate. In total, 600 MCP genes were detected across 88 assembled transcriptomes in a range of eukaryotic groups, similar to the genomic dataset. These included virophage-type EVEs (MMseqs protein clusters 45 to 46; *SI Appendix*, Table S6), which were found in chrysophyte, dinoflagellate, and amoebozoan transcriptomes, among others, and PgVV-like MCP genes (MMseqs protein clusters 86 to 87), which were found in haptophyte transcriptomes. Although there was no transcriptome available for *P. micropora*, we found 20 similar MCP genes (protein clusters 100, 32, 11; *SI Appendix*, Table S6) in the transcriptome from the related *P. chromatophora*, suggesting that members of several different virus groups are expressed in this organism. Notable new protist groups expressing Maverick–Polinton-type MCP genes came from a number of parasitic alveolates in the Class Conoidasida that usually inhabit the gut of nematodes and mollusks. Because this group of MCP genes is almost exclusively associated with animals, this indicates either host contamination of the transcriptomes or horizontal transfer of elements into these organisms.

## Discussion

Our comprehensive genomic survey revealed that 14 to 40 kbp long DNA viruses of the Polinton-like supergroup are frequent inhabitants of diverse protist genomes. Moreover, our finding of thousands of endogenous viruses in one-third of all sequenced protist genomes may still be a conservative estimate. A multitude of factors have conspired to hitherto hide most endogenous viruses in protist genomes from detection. First, sequencing bias against low-GC DNA causes a reduced yield for Illumina data; hence, endogenous virus regions are sometimes underrepresented in assemblies, as shown for the mavirus virophage (38). Second, protist EVEs are often present in high copy numbers, with hundreds or thousands of microvariants, which causes genome assembly problems from short-read data and in turn leads to artificially fragmented and poorly annotated genes due to false consensus sequences. Third, during sequence submission and processing, many contigs with endogenous viruses, especially those that assemble separately, may be removed during automated decontamination pipelines. Finally, low sequence conservation makes many protist EVEs difficult to identify, unless sensitive methods for remote homology detection or computationally expensive protein structure prediction are applied. The increasing use of accurate long-read sequencing alleviates many of these methodological problems, allowing for easier detection of endogenous viruses in protist genomes and revealing the true scale of their eco-evolutionary impact.

Our study has identified eukaryotic hosts for most PLV groups detected previously in environmental viromes, which were predicted to be free viruses (17). Combined with evidence from long-read sequencing (Fig. 3) and protist transcriptomes (*SI Appendix*, Table S6), this suggests many EVEs may be functional. The major outstanding question is now what functional role they play? As they represent multiple virus groups, they may differ in their origins and lifestyles. Some could be lysogenic viruses, as predicted for TsV-N1, which were previously found as both EVEs in *Tetraselmis* spp. and viral particles infecting *Tetraselmis striata*. The possibility also remains that some EVEs may be capable of replication by transposition. Polintons were originally predicted to be transposons before viral capsid genes were detected, and many EVEs we have discovered here lack a detectable DNA polymerase gene, which could indicate that they use an alternate mode of replication. However, in our dataset, we observed that DNA Helicase domains were often fused to an unknown domain. Therefore, it is possible that we are not yet able to detect the replication enzyme in these elements. EVEs within a genome tend to possess related MCP types with sometimes hundreds of variants coexisting. This suggests that replication within a particular genome or protist group is more common than horizontal transfer between groups. Such observations could be caused by both intragenomic transposition, and by actively replicating exogenous viruses that repeatedly integrated into their host genomes. Because all entities in our study possess an MCP gene, which would be expected to be lost if they were strict transposons, it is possible that some elements may be capable of a dual lifestyle, with both viral and transposon-like replication taking place. Finally, there is growing evidence that some groups of EVEs may possess virophage-like lifestyles, requiring a coinfecting giant virus for their activation and replication. The PgVV group virus Gezel-14T co-replicates only in the presence of a giant virus (21), despite lacking detectable sequence similarity to bona fide virophages of the *Maveriviricetes* class. PgVV group members are found as EVEs throughout chlorophytes, suggesting that this strategy may be more widespread. We also find similarities between the Gossevirus group of PLVs and the *Maveriviricetes* virophages. Gosseviruses possess all the core genes of virophages including a cysteine protease gene, and their modeled MCP structure is most similar to the Sputnik virophage MCP, as opposed to MCPs from other Polintons and PLVs, which are more similar to NCLDV capsid proteins (*SI Appendix*, Tables S2 and S3). Even if only some of the EVE groups described here interact with giant viruses, the ecological significance would be paramount. The existence of tens to thousands of related, but nonidentical, EVEs in a single protist genome could imply that much of the diversity of giant viruses is countered by similarly diverse host responses in the shape of active endogenous viruses. We expect that detailed experimental studies of individual virus–host systems will reveal a wide range of functions for these variable and abundant eukaryotic EVEs. Taken together, our findings provide an exciting perspective into the intricate and complex virus–host arms races that have shaped evolution across the eukaryotic tree of life.

## Materials and Methods

### Building the Virus MCP Database.

Polinton-like virus and virophage MCP genes described in Bellas and Sommaruga (17) were clustered at 30% identity across 70% length using MMseqs2 (33). Cluster representatives (referred to henceforth as “PLV MCP reps”) were used to iteratively interrogate GenBank WGS protist assemblies to detect distantly related MCP genes as follows: I) All protist assemblies in GenBank Whole Genome Shotgun (WGS) were downloaded in December 2021 (1,352 genomes) using Entrez Direct (EDirect) by selecting for taxid 2,759 (Eukarya) and excluding taxids: 4,751 (Fungi), 33,208 (Animalia), and 3,193 (higher plants). Records that were generated from Metagenomic Assembled Genomes were excluded, except in the case of *Streblomastix strix*, which used a nonstandard translation table (*SI Appendix*, Table S6) and allowed us to be confident that virus sequences arose from within the host genome. II) Genes were predicted on all GenBank WGS assemblies using MetaGeneMark (43). Using such a prokaryotic/virus gene prediction tool works well for endogenous viruses as they do not contain introns and can be predicted rapidly. The exception was *S. strix* which was dealt with separately. III) To reduce the database size for searches, gene predictions were filtered for amino acid sequences of between 200 and 900aa in length, before being clustered at 50% identity across 80% length using MMseqs2 (easy-cluster--min-seqs-id 0.5 -c 0.8). IV) To detect distantly related MCP genes, an iterative search of the above gene clusters was conducted with JackHMMER (HMMER 3.1b2). The PLV MCP reps were each queried against the clustered WGS database (step III) using five iterations of JackHMMER (-N 5 -E 1e-7). To facilitate the building of the HMM profiles in the initial iteration, the WGS database (step III) was also seeded with all MCP genes from Bellas and Sommaruga (17). V) To determine nonstandard genetic code MCP hits in *S. strix*, *T. vaginalis* MCP hits were used in an online PSI-BLAST search of the GenBank nonredundant protein database (nr) (E-value cutoff 10^−5^) (https://blast.ncbi.nlm.nih.gov/Blast.cgi). Matching *S. stri*x records were downloaded as amino acid sequences and confirmed as below. VI) To confirm whether the hits from JackHMMER belonged to virus MCP genes, hits were clustered and subjected to further checks. All candidate MCP genes were clustered with MMseqs2 at 30% identity across 80% length, and each cluster was aligned with MAFFT v7.490 (44) with the alignment used as an input to HHpred (https://toolkit.tuebingen.mpg.de/tools/hhpred) (settings: global:realign). Hits were then characterized as viruses where the best hit was to a double jelly-roll (DJR) fold virus MCP gene (*SI Appendix*, Table S2). When no HHpred hit could be found, ColabFold (34) was used to predict the protein structure using the MAFFT cluster alignment (step VI) as a multiple sequence alignment (MSA) input. The top model prediction was uploaded to Foldseek (36) (https://search.foldseek.com/search) to compare against structure predictions from PDB100 using the 3Di/AA mode. If the top hit was to a double jelly-roll fold viral MCP gene, we considered this a confirmed MCP gene (*SI Appendix*, Table S3). VII) All confirmed MCP genes were added to all MCP genes from Bellas and Sommaruga (17) to create a final confirmed MCP database from Maverick–Polintons, PLVs, and virophages to be used in further analysis. Finally, to group MCP genes from the database into protein clusters for *SI Appendix*, Table S4 and Fig. 1, all MCP genes were clustered with MMseqs2 at 25% identity across 30% length, defining 121 MCP clusters.

### Searching All GenBank WGS Protist Assemblies for MCP Genes.

DIAMOND BLASTX (37) was used to search every WGS contig from every genome assembly against the confirmed MCP database. We allowed for frameshifts to detect degraded sequences, sequencing errors or nonstandard translation tables, and used range culling to limit the results to one hit per query range, so multiple hits could be detected per contig, but only one hit per individual MCP gene (settings --evalue 1e-12 --range-culling -F 15 --max-target-seqs 1). The resulting output was then filtered to a minimum of 23% identity across a 100aa length to create a table of confirmed MCP matches (*SI Appendix*, Table S5). Of these, representatives from 73 MCP clusters were found integrated into protist genomes, while 48 MCP clusters were only found in environmental metagenomes.

### TSA Virus MCP Genes.

All assembled protist transcriptomes from GenBank TSA were downloaded using EDirect as before. Transcriptomes were screened for MCP genes using DIAMOND BLASTX as above, but with a minimum sequence length of 50 amino acids to account for the shorter contigs.

### Annotation of MCP Loci and Inspection with gggenomes.

To further investigate whether detected MCP genes correspond to EVEs, we analyzed and visually inspected their genomic context. We extracted up to 30 kbp up and downstream of every MCP hit from the host contigs and annotated them for viral features. We used seqkit (45) for sequence data manipulations (extraction, conversion, translation), bedtools (46) to compute GC contents along sequences, and MMseqs2 (easy-search -s 7.5 --greedy-best-hits 1) to align MCP loci to known virophage and PLV hallmark proteins. The hallmark proteins were derived from proteomes of over 1,000 previously published virophages and PLVs which we downloaded from NCBI or sources listed in the respective publications (17, 19, 25–27, 31, 38, 47–55). Proteins were annotated with Prodigal v2.6.3 and clustered with MMseqs2 (-s 7 -e 1e-4 -c 0.7). Functional annotations for each cluster were propagated based on either members with known functions or hits among clusters based on remote homology detection with HHsearch (56) combined with manual curation based on comparisons of multiple sequence alignments. The hallmark protein database was created by retaining all clusters with known functions associated with a viral lifestyle and clusters conserved across many of the reference genomes (conserved hypotheticals). To annotate MCP loci, the hallmark clusters were converted into an MMseqs2 profile database, and loci were aligned against it. TIRs were annotated with a modified version of Minimap2 (57), which can be restricted to only report self-mappings, with parameters optimized for short high-identity hits (-S –rev-only -c -m 30 -n 3 -c -B5 -O6 -E3 -k 10 -s 60). The annotated MCP loci were visualized with gggenomes v0.9.5 (https://github.com/thackl/gggenomes).

### Read and contig Polishing.

We downloaded PACBIO raw reads from *Pedospumella encystans* strain JBM/S11 (SRR9203571) and *Synura* sp. LO234KE (SRR9203568) and screened these for viral MCP genes using DIAMOND BLASTX as above. Contigs with confirmed MCP genes were extracted and polished with publicly available Illumina short-read data (SRR9203575 for *Synura* sp. LO234KE and SRR9203573 for *P. encystans*). Polishing was performed in Geneious Prime 2022.2.2 (www.geneious.com) by mapping Illumina reads using the Geneious mapper (Settings: Custom sensitivity; Maximum 10% gaps per reads, Word Length 18, Maximum Mismatches per read 10%, Max Gap size 10; Only map paired reads which map nearby; Iterate up to 20 times). A large 10% mismatch error was allowed to account for PACBIO read error in this dataset. Genes were predicted using Glimmer (Genetic code 11) and annotated against the Protein Data Bank (PDB) using HHpred (https://toolkit.tuebingen.mpg.de/tools/hhpred; Settings: HHblits=>UniRef30. global:realign).

For *Hydrurus foetidus,* most MCP hits were on short fragments. Only one contig was long enough to show a complete EVE; however, the contig contained numerous stop codons. To improve this, we downloaded the longest contig (GenBank UYFQ010000146; 22 kpb) and polished it using the Geneious mapper and strict mapping criteria (Maximum 5% gaps per read, Maximum Mismatches 5%, Only map paired reads which map nearby, Maximum gap size 6; Iterate 2 times). Genes were predicted with Glimmer and annotated with HHpred as above.

### Long-Read Sequencing.

Based on our DIAMOND BLASTX searches above, we determined that the chrysophyte *Dinobryon sp.* and Synurid *Synura* sp. LO234KE contained multiple hits to EVE MCP genes. To test whether these hits represented EVEs within a genome, we sequenced the two genomes using long-read technology. Cultures were obtained courtesy of the laboratory of J. Boenigk (University Duisburg-Essen, Germany). Nonaxenic cultures were grown in WC medium under a light/dark cycle of 12:12 h at 16 °C. To harvest, cells were pelleted at 1000× g, twice washed in WC medium, and again pelleted, before DNA extraction using a DNeasy PowerWater Kit (Qiagen). Nanopore libraries were prepared using the Ligation Sequencing Kit (SQK-LSK-110) and sequenced on an R10.4 flow cell. Basecalling was done using the Super Accuracy mode in Guppy. All short reads (<5 Kbp) were removed, resulting in 1 Gbp of raw data for *Dinobryon sp.* (103,571 reads; N50 of 9,910; max length 126,523) and 1.2 Gbp data for *Synura* sp. (105,081 reads; N50 of 12,177; max length 123,019). These reads were assembled using Flye v2.9 (58) (settings --nana-hq --meta -g 100m --read-error 0.03 --iterations 3). A second assembly was carried out on *Synura* sp. using reads >10 kbp (49,447 reads) to maximize the chance of successful EVE assembly. Selected EVEs were polished using Illumina reads (SRR9203575 for *Synura* sp. LO234KE and SRR9203574 for *Dinobryon* sp. L0226KS) using the Geneious Prime read mapper (Maximum 5% gaps per read, Maximum Mismatches 5%, Only map paired reads which map nearby, Maximum gap size 6; Iterate 15 times).

### Analysis of Within-Species MCP Diversity.

To analyze the diversity of EVE MCP genes within a single organism, the genome of *P. micropora* (WGS Accession BJOX01) was chosen. The DIAMOND BLASTX hit coordinates were used to extract genomic regions corresponding to MCP gene hits before nucleotide sequences were clustered using MMseqs (50% identity across 30% length) to form five main clusters which were individually aligned using MUSCLE v3.8.1551 (59). Maximum likelihood trees were constructed using PhyML v3.0 (60) and visualized using ITOL (https://itol.embl.de/).

### Network Analysis of Endogenous MCP Genes from Protists.

We attempted to retrieve all matching MCP genes from protist genomes. First, the DIAMOND BLASTX output was converted into a bedfile and these coordinates were then used to retrieve the corresponding region from the assembly files using bedtools (61). To reduce redundancy, MCP genes were individually clustered in each genome at 90% identity (MMseqs settings -c 0.8 --min-seq-id 0.9). Representative sequences from each cluster, in each genome, were translated into amino acid sequences in the correct reading frame by predicting genes with MetaGeneMark (-m MetaGeneMark_v1.mod). Amino acid sequences over 200aa in length were kept for further analysis, hence incomplete, or sequences with multiple frameshift errors were discarded for network analysis. In the case of *S. strix*, the bedfile regions were extended by 50 bp on either side before bedtools extraction. Gene prediction, in this case, was by Prodigal v2.6.3 (62) with translation Table 6 set (settings: -g 6). To complete the network analysis, all Maverick–Polinton, Adintovirus, vertebrate Maverick, and metagenomically detected MCP genes (13–15, 17) were added to the MCP file. To reduce the complexity of the analysis and resulting network, vertebrate Maverick–Polintons were first clustered at 90% nucleotide identity and metagenomically detected MCPs at 70% amino acid identity. The Enzyme Similarity Tool (EFI-EST https://efi.igb.illinois.edu/efi-est/) (63) was used to create a sequence similarity network using all-vs-all blast searches (1e-4 cutoff) and a minimum alignment score of 6. The network was visualized in cytoscape using a prefuse force-directed layout.

## Supplementary Material

Appendix 01 (PDF)

Appendix 02 (XLSX)

Dataset S01 (PDF)

## Data Availability

Supplementary raw data are available at https://doi.org/10.6084/m9.figshare.21581355.v3 (39) and comprise: 1) AlphaFold structural predictions (.pdb) of MCP genes; 2) all MCP genes confirmed by HHpred or Alphafold (Fasta format); 3) Cytoscape network of MCP genes used in Fig. 4 generation; 4) MCP genes retrieved from all protist genomes; 5) high-quality EVEs (7382) in protist genomes and associated annotation tracks; 6) nanopore assemblies and PLV containing contigs from *Synura* sp. and *Dinobryon sp.* GenBank WGS accession numbers and coordinates to EVE MCP genes in all protist genomes can be found in *SI Appendix*, Table S5. Contigs can be retrieved from https://www.ncbi.nlm.nih.gov/Traces/wgs/?view=wgs.
