# Supplementary material for: Large-scale invasion of unicellular eukaryotic genomes by integrating DNA viruses
Source: Proc Natl Acad Sci U S A. 2023 Apr 10;120(16):e2300465120. doi: 10.1073/pnas.2300465120 (PMC10120064; doi:10.1073/pnas.2300465120)
Supplement: Supplementary file 1 — Appendix 01 (PDF) [file pnas.2300465120.sapp1.pdf]

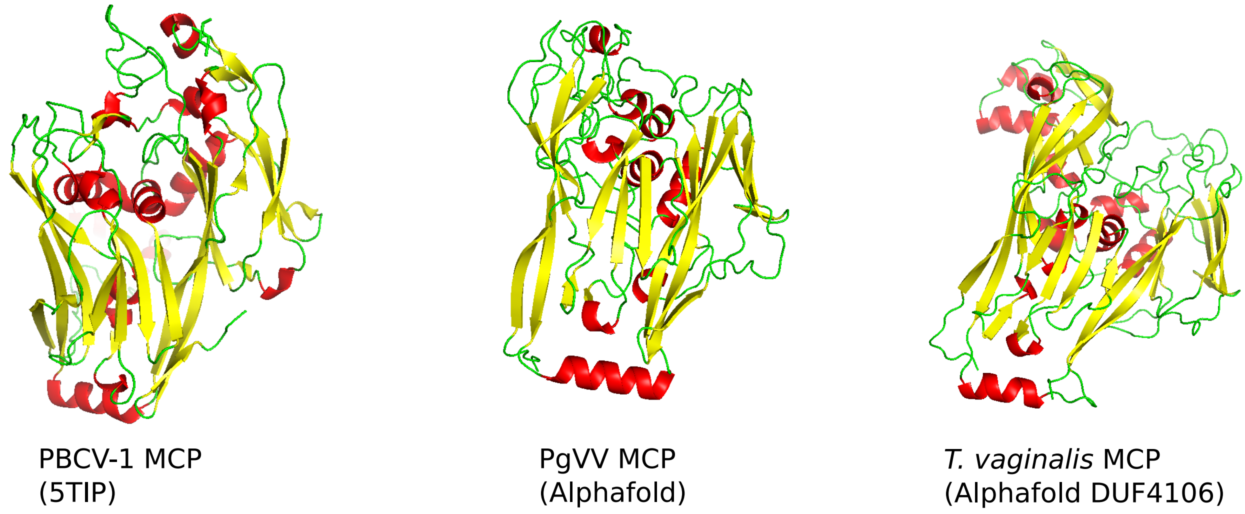

Fig. S1. Predicted major capsid protein structures of PgVV and *Trichomonas vaginalis* Polintons. The AlphaFold structure predictions of the PLV PgVV and the MCP type found in *T. vaginalis* are comparable to the experimental MCP structure of the large DNA virus PBCV-1 (RSCB Protein Data Bank entry 5TIP), suggesting the former are double-jelly roll capsid proteins. Model colors: red - alpha-helices; yellow - beta-strands.

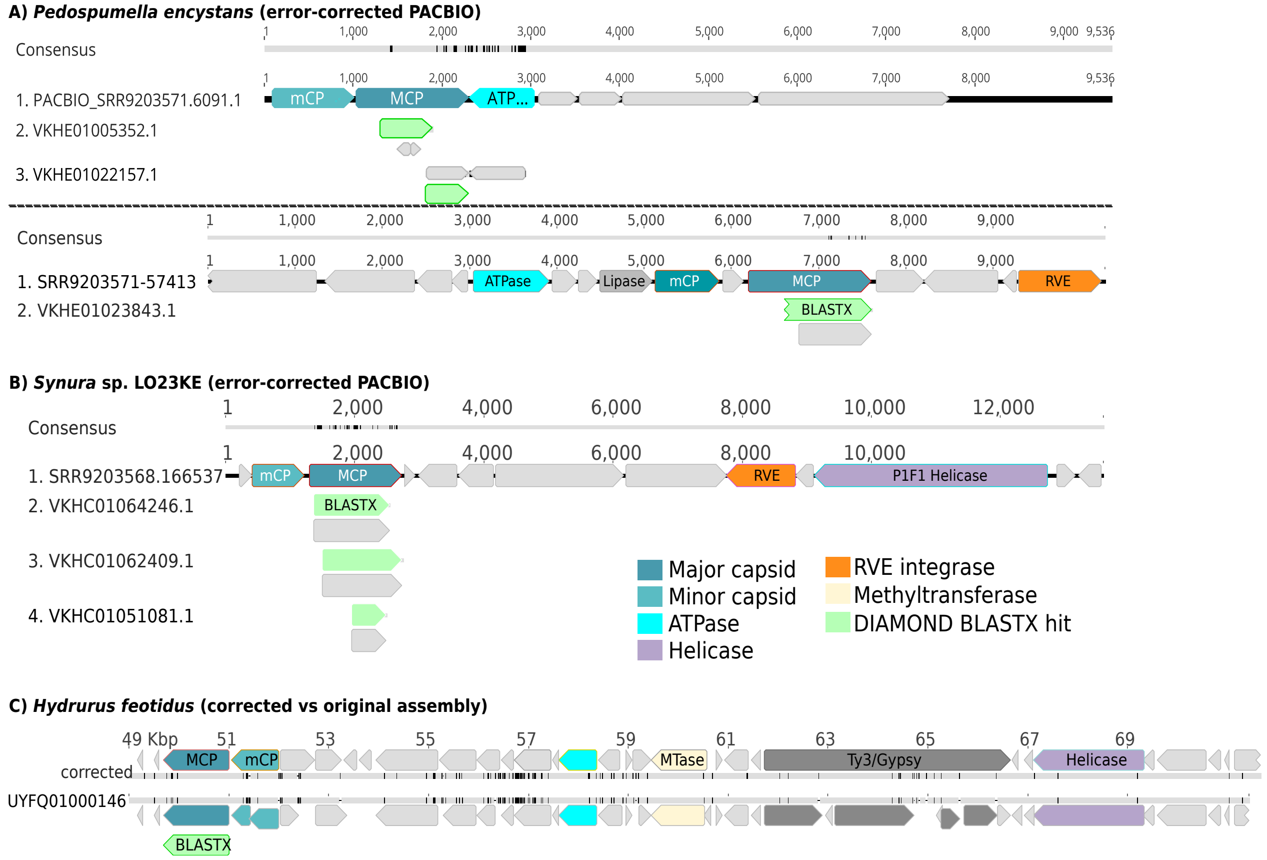

Fig. S2. Error-correction of long-read sequences with Illumina data reduces fragmentation of endogenous viral genes. (A) *Pedospumella encystans* strain JBM/S11 and (B) *Synura* sp. LO23KE PACBIO reads of ~10 kbp were error-corrected using Illumina short reads (Methods) before gene prediction. The published GenBank WGS assemblies (VKH prefix) from which we detected MCP genes (green annotation showing DIAMOND BLASTX hit from Table S5) were mapped to the polished PACBIO reads (SRR prefix) from the respective organism. Note: Multiple closely related, but not identical, MCP gene assemblies from GenBank WGS could be mapped to each contig, showing how such closely related genes cause problems for short-read C) *Hydrurus foetidus* contig from GenBank WGS (UYFQ010000146; assembled from Illumina reads) aligned with a polished version of the same contig (Methods).

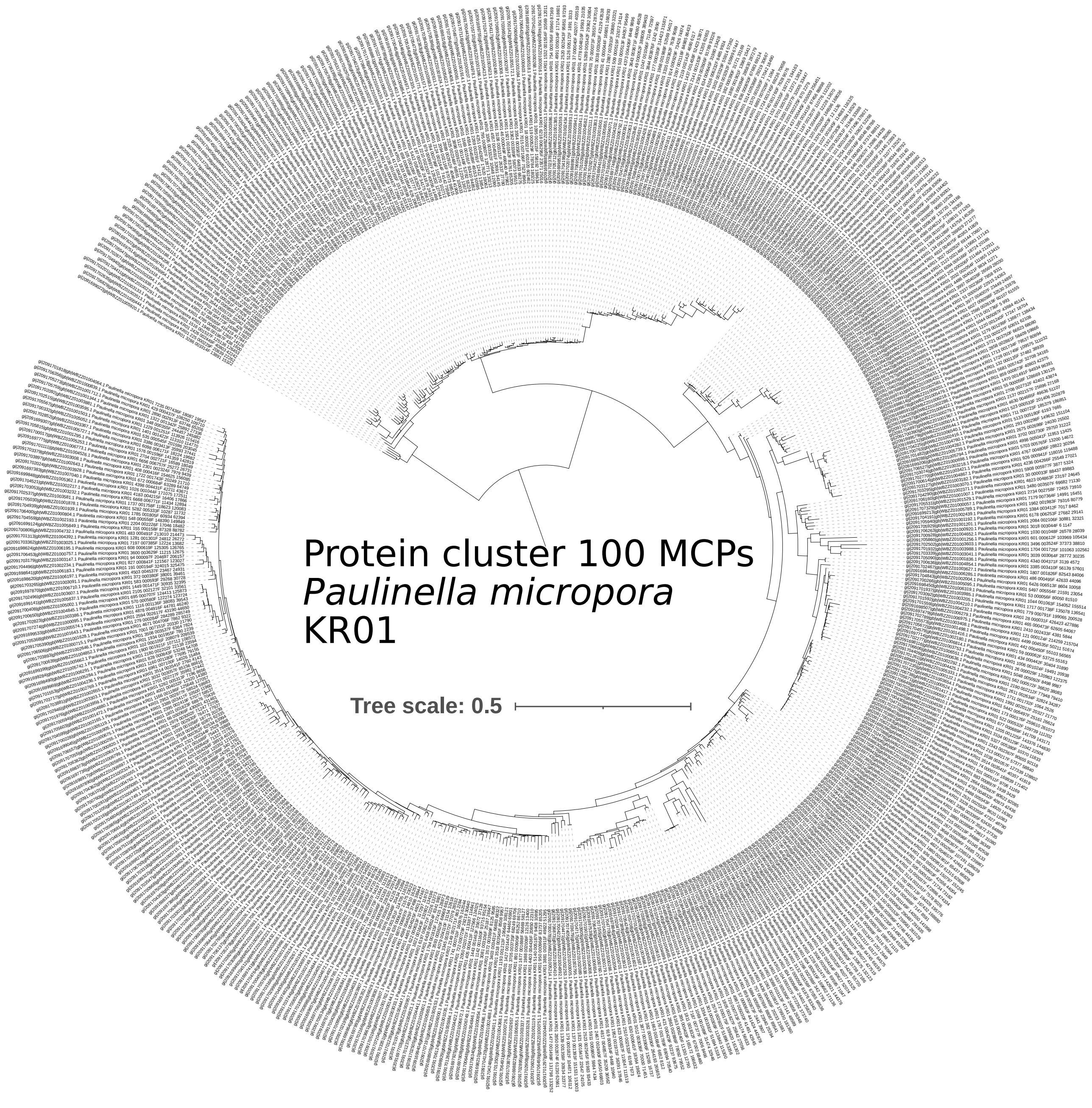

Fig. S3. Maximum likelihood tree of MCPs retrieved from *Paulinella micropora* KR01 that belong to MCP protein cluster 100.

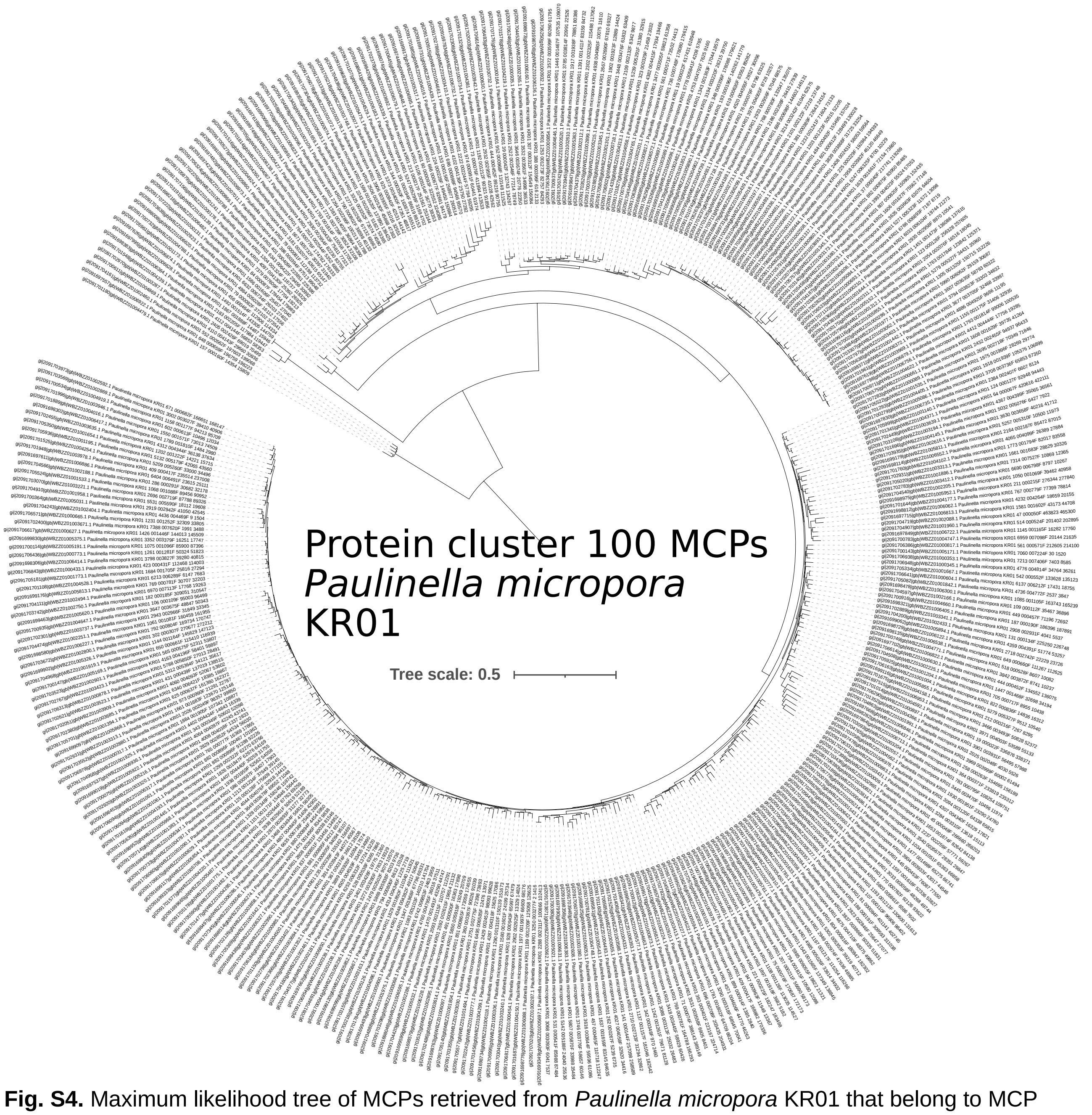

Fig. S4. Maximum likelihood tree of MCPs retrieved from *Paulinella micropora* KR01 that belong to MCP protein cluster 100 (part 2).

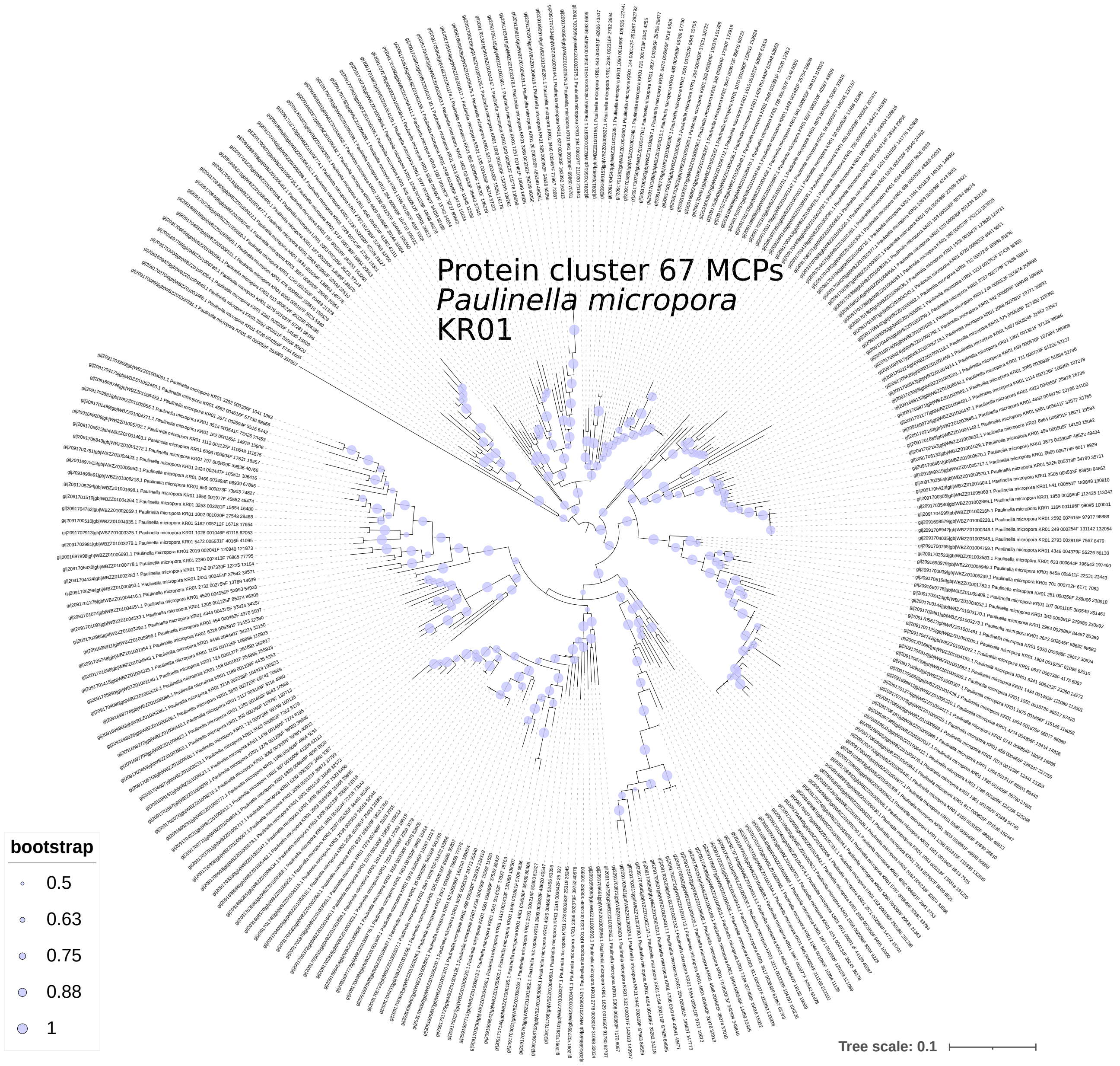

Fig. S5. Maximum likelihood tree of MCPs retrieved from *Paulinella micropora* KR01 that belong to MCP protein cluster 67.

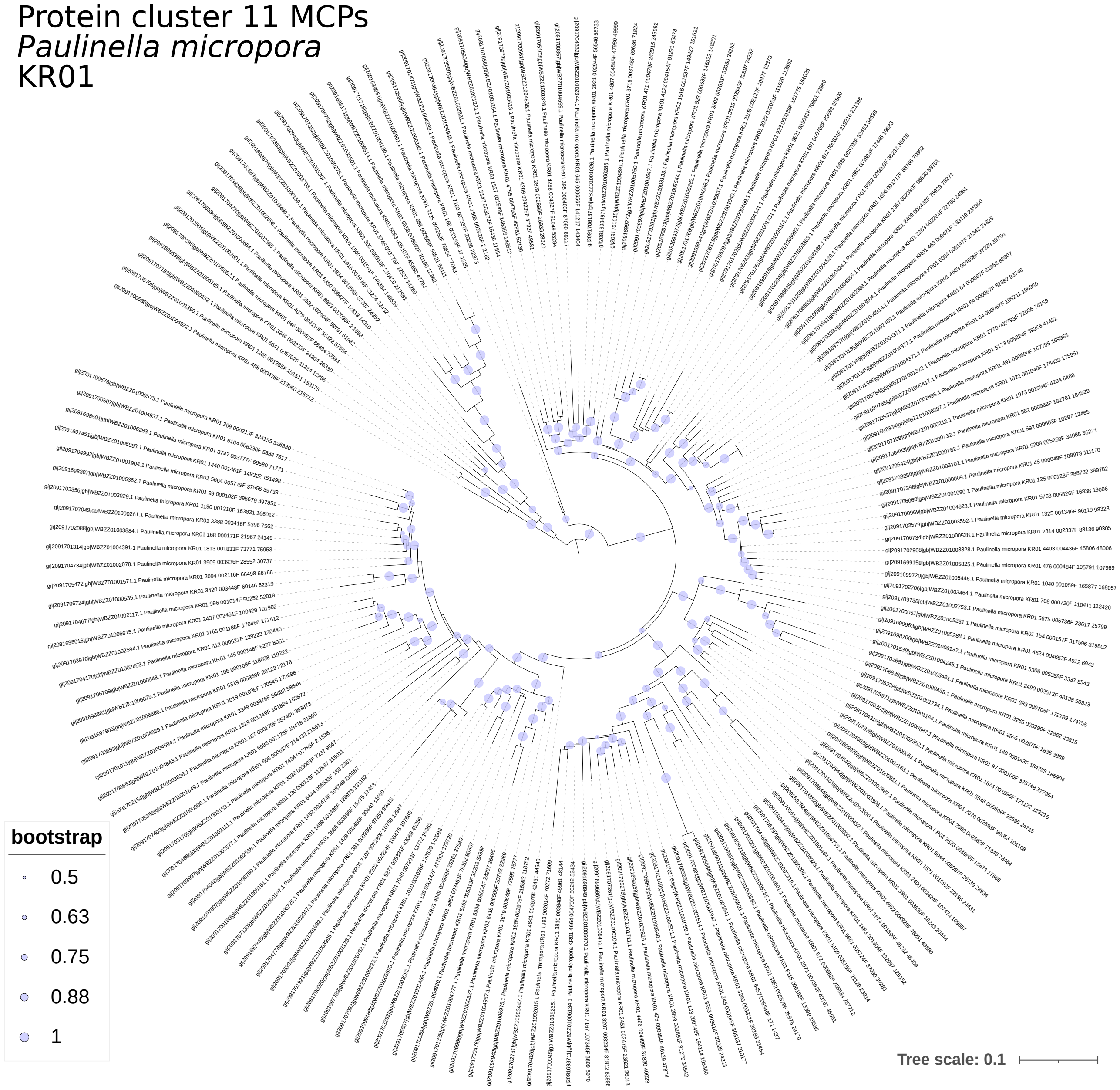

Fig. S6. Maximum likelihood tree of MCPs retrieved from *Paulinella micropora* KR01 that belong to MCP protein cluster 11.

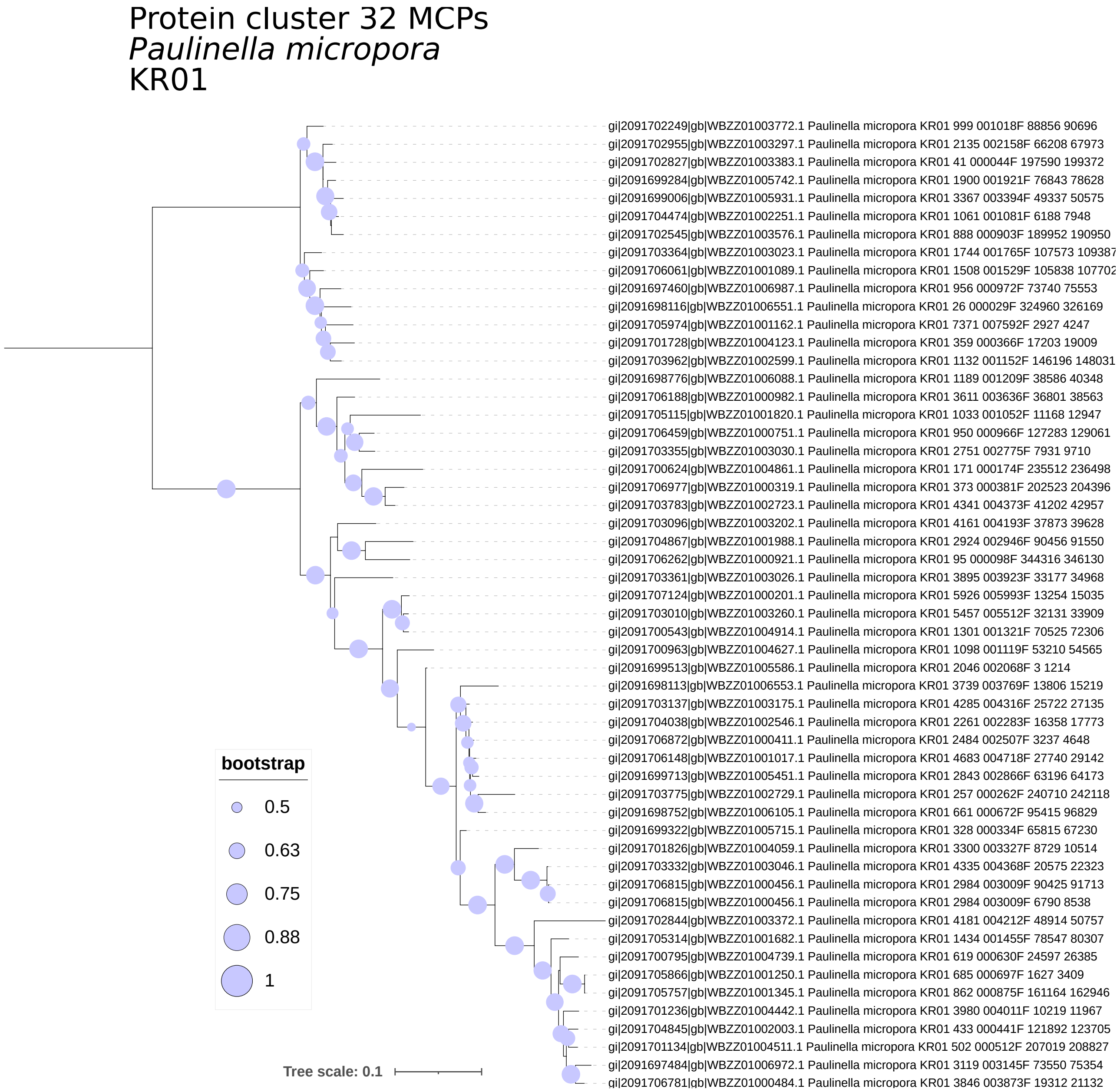

Fig. S7. Maximum likelihood tree of MCPs retrieved from *Paulinella micropora* KR01 that belong to MCP protein cluster 32.

Supplemental information

Table S1. WGS genomes analysed (Dec 2021). All downloaded and analyzed WGS files.

Table S2. HHpred confirmation of MCP genes.

Table S3. Colabfold and Foldseek MCP detection. Foldseek confirmation of MCP genes based on Colabfold pbd models.

Table S4. MCP Protein clusters detected in each protist genome. PLV, Maverick-Polinton and virophage MCP protein clusters detected in each protist genome from GenBank WGS (DIAMOND BLASTX 1e-12, 100aa length, 23% identity minimum). MCP Clusters (MMPCs) are denoted on row 6 and were generated by clustering all confirmed MCP genes at 25% identity across 30% length with MMseqs).

Table S5. DIAMOND BLASTX raw MCP search results. Each WGS contig is DIAMOND BLASTX searched against all MCP genes. Column F is full GenBank accession, column Q and R are the start and end nucleotide position of the hit. Each MCP should produce 1 hit, but multiple MCP genes can sometimes be detected on the same contig.

Table S6. MCP genes in transcriptomes (GenBank TSA). Each transcriptome assembled contig is screened for MCP genes.

Dataset 1. Genome maps of EVEs in every organism. Additional genome maps of the top five largest EVEs from every protist genome in this study.
