# Supplementary figures and images for: Large-scale invasion of unicellular eukaryotic genomes by integrating DNA viruses
Source: Proc Natl Acad Sci U S A. 2023 Apr 10;120(16):e2300465120. doi: 10.1073/pnas.2300465120 (PMC10120064; doi:10.1073/pnas.2300465120)

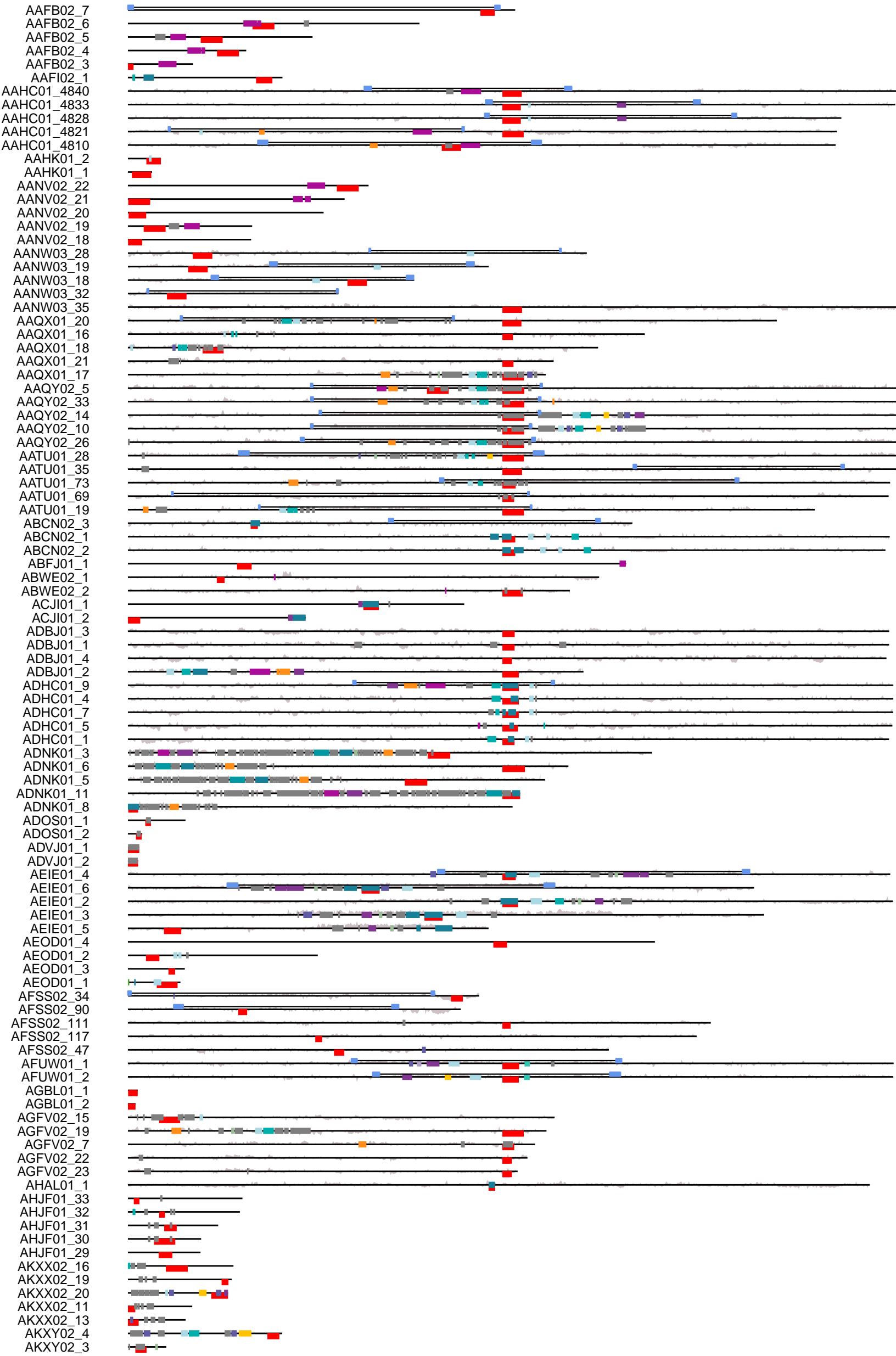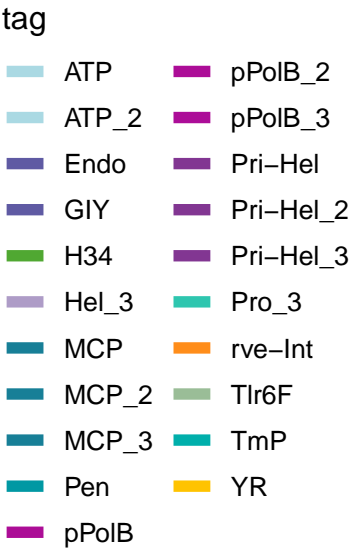

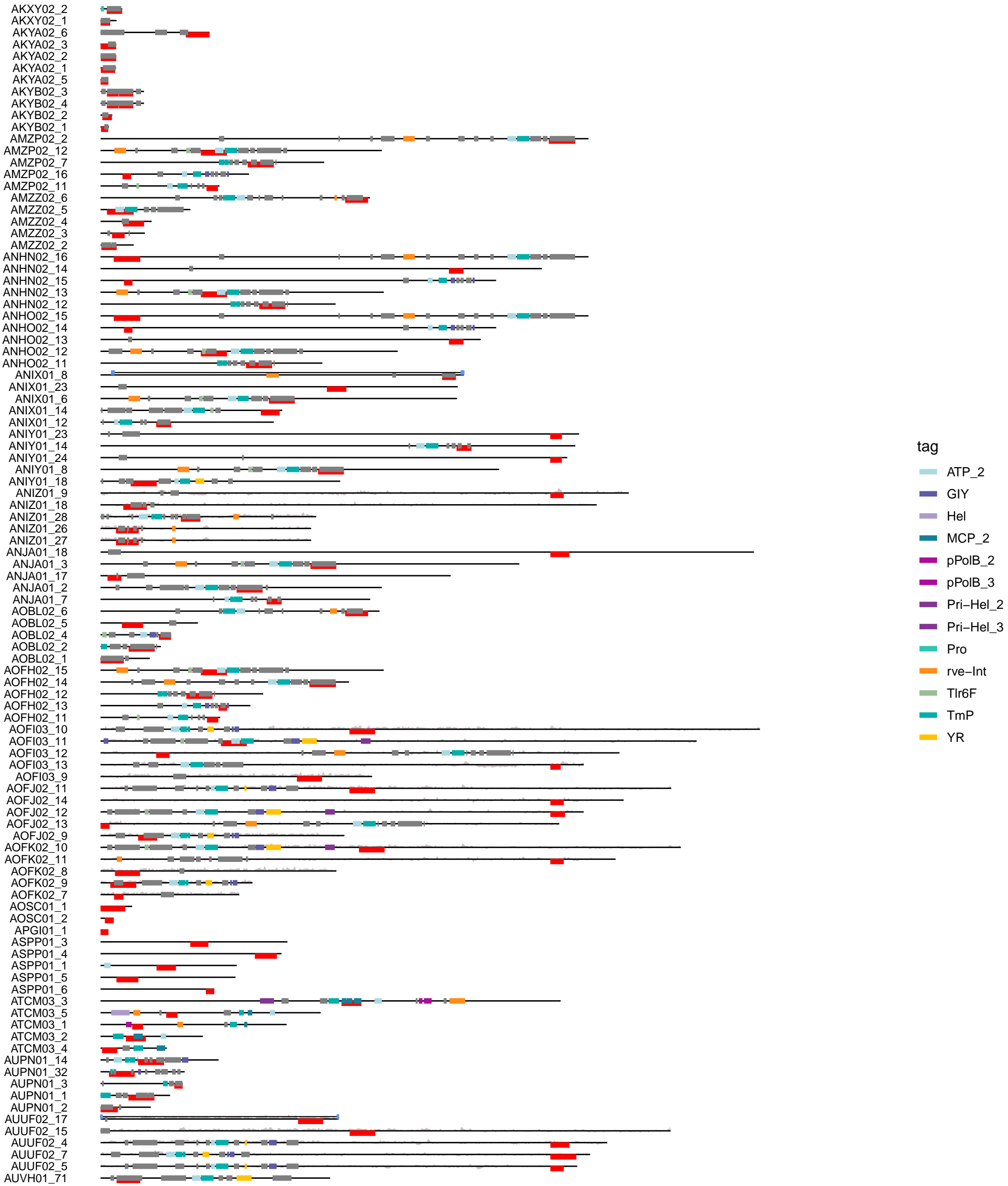

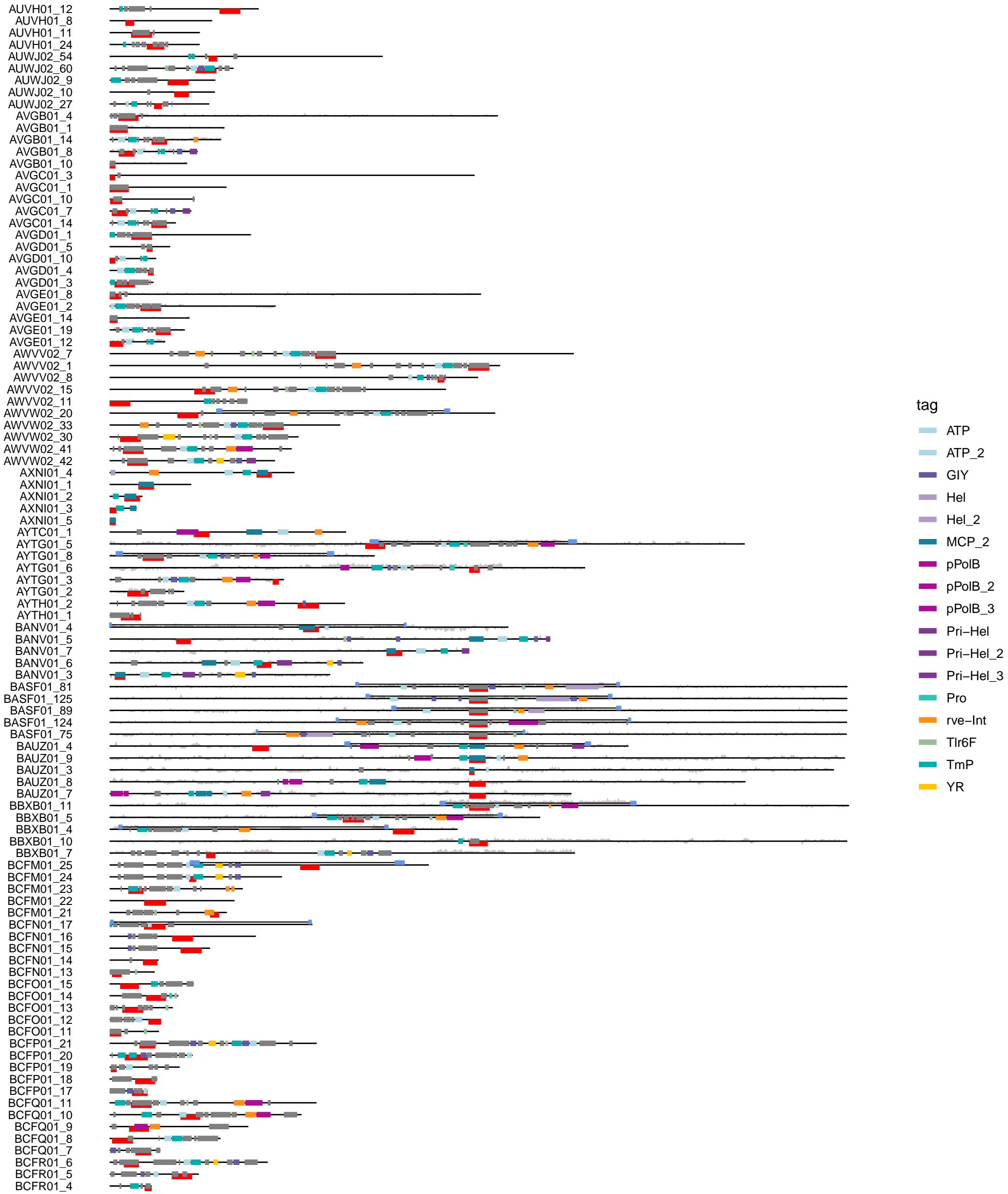

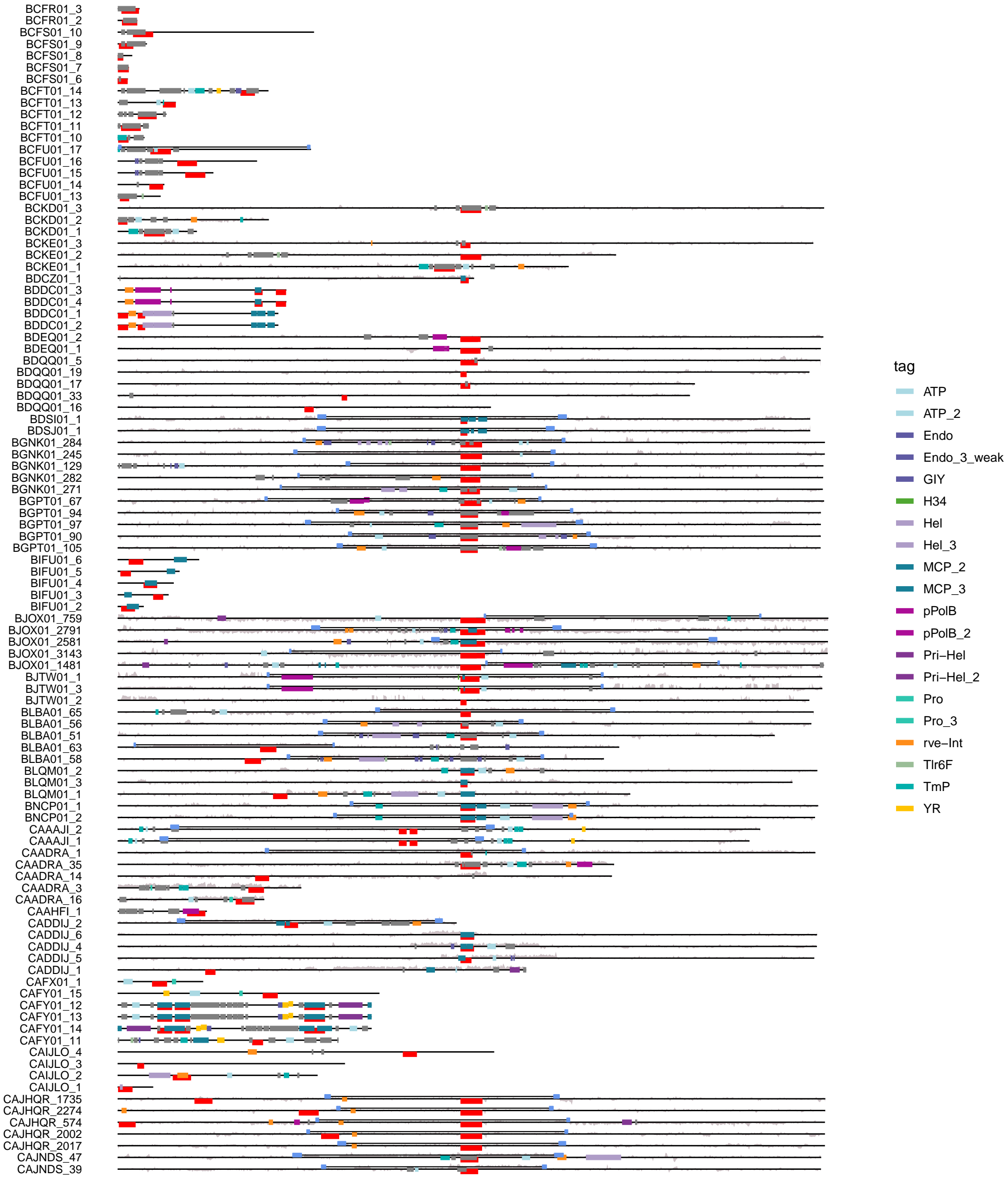

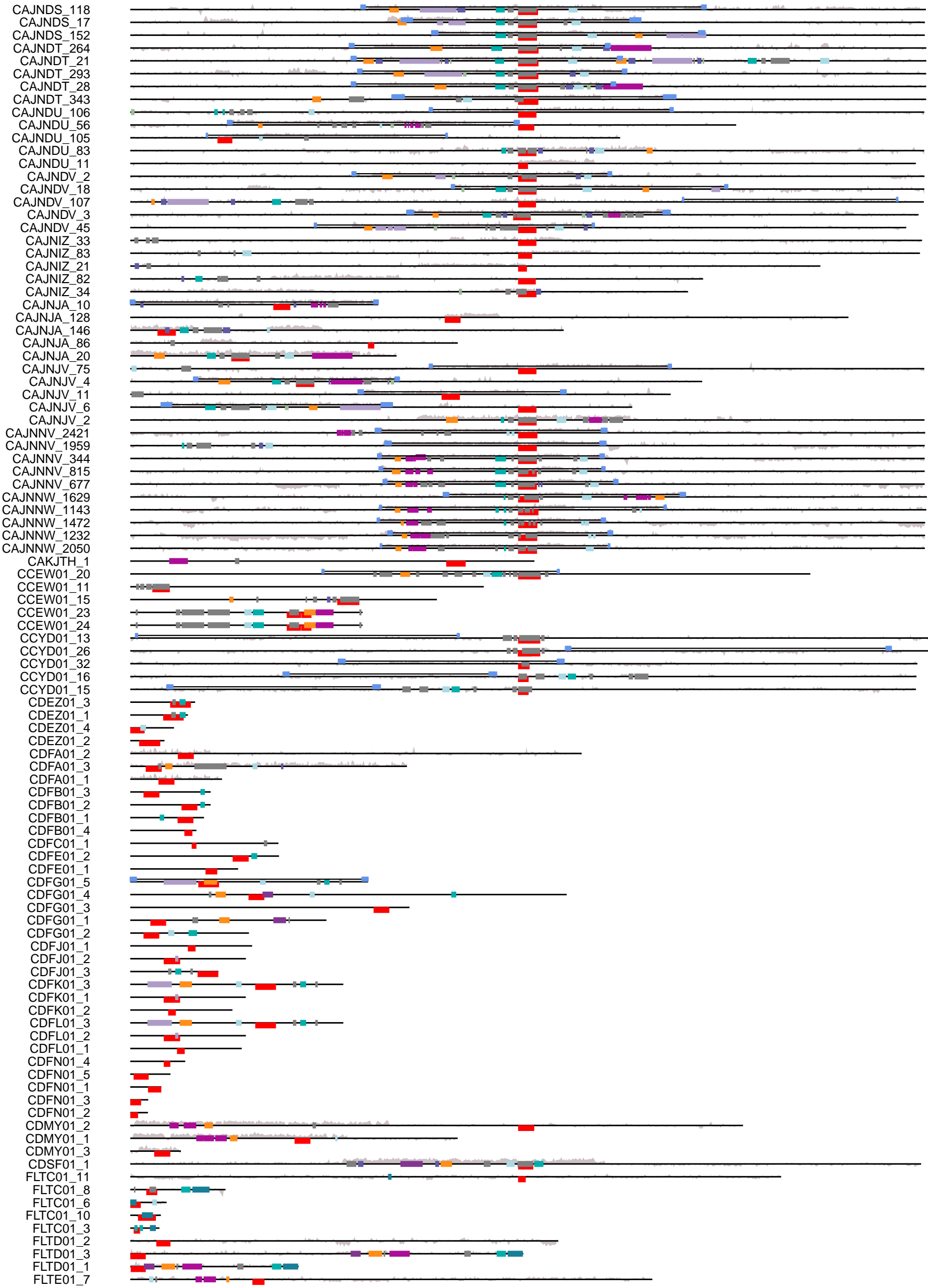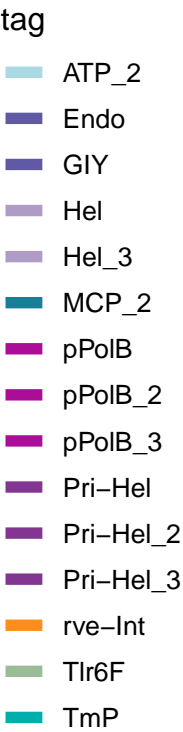

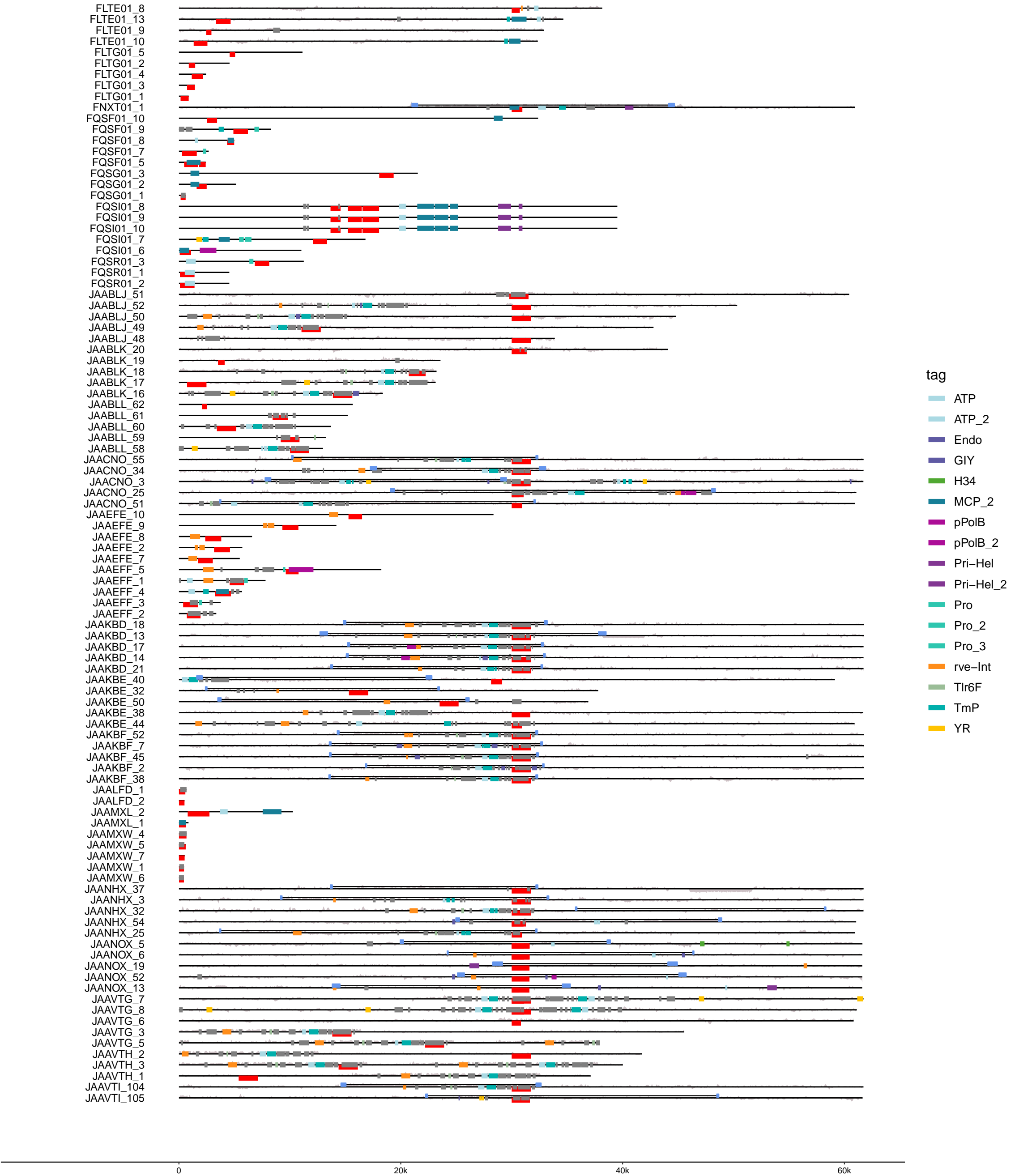

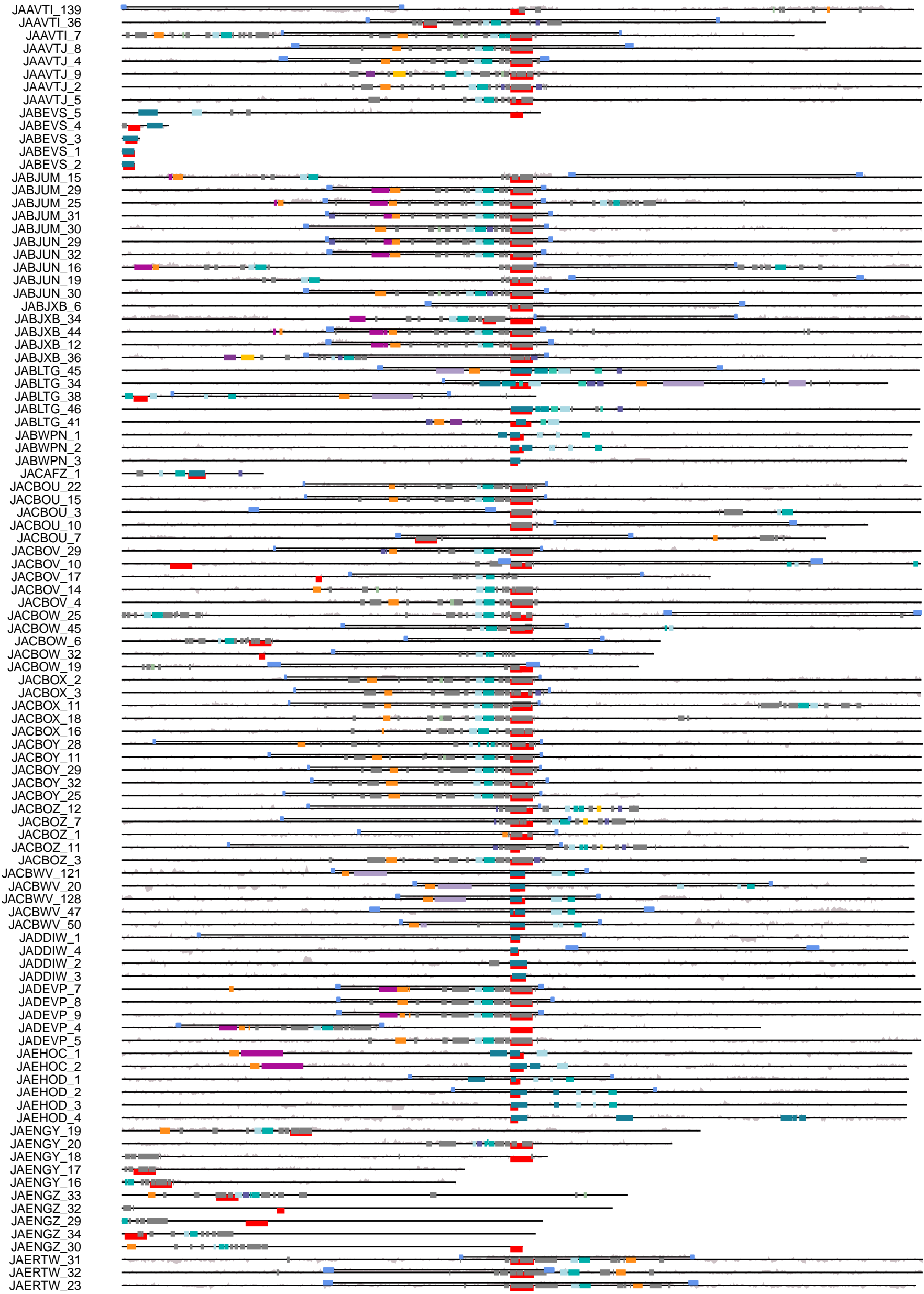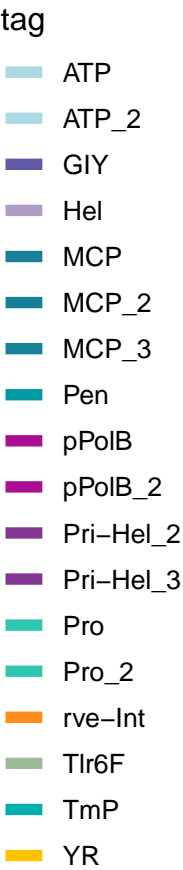

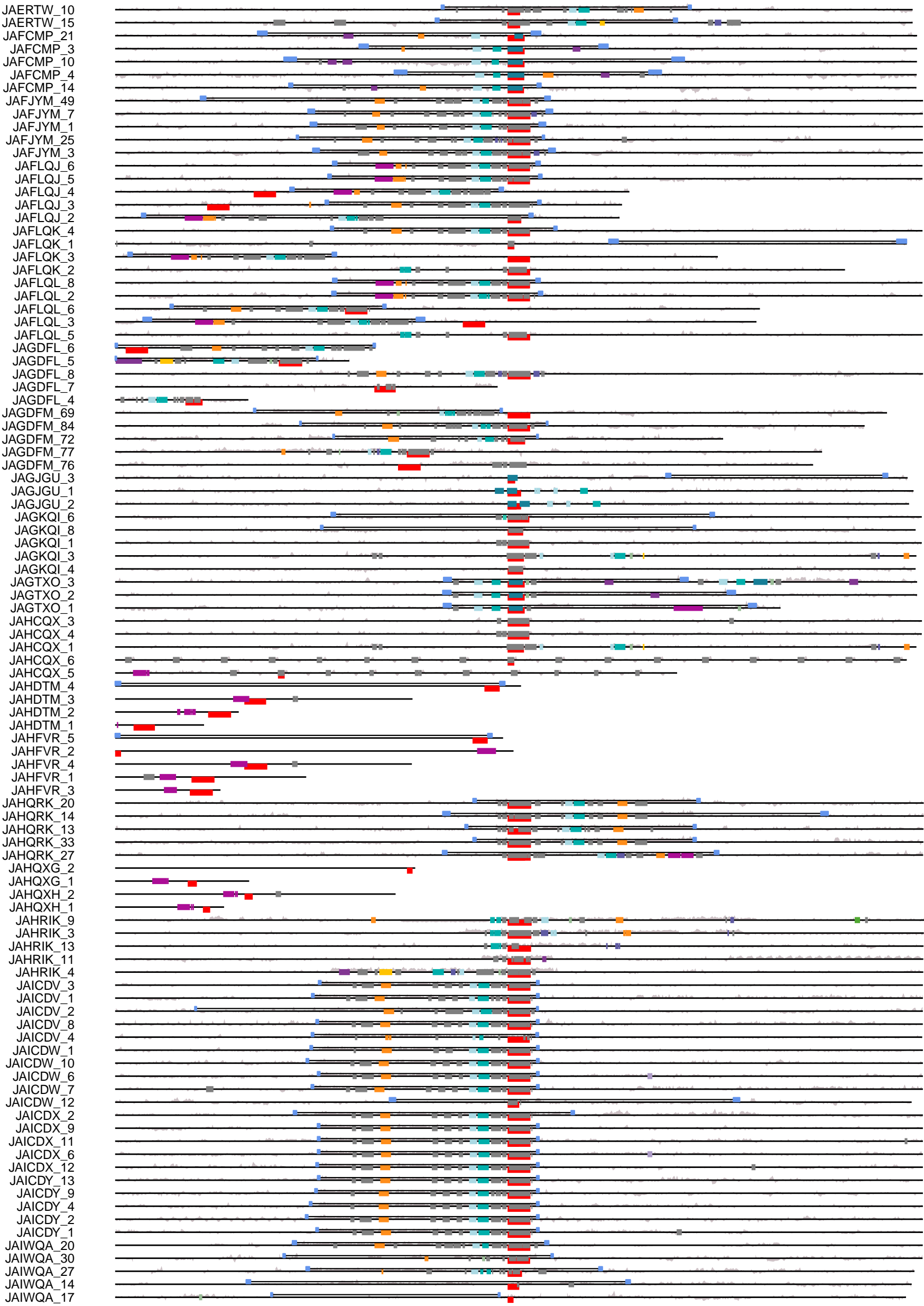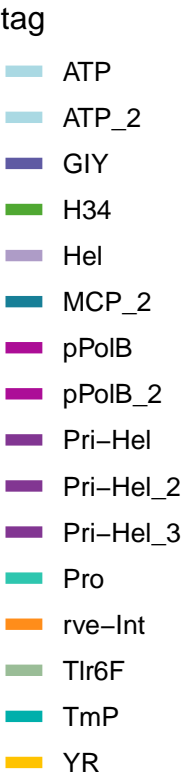

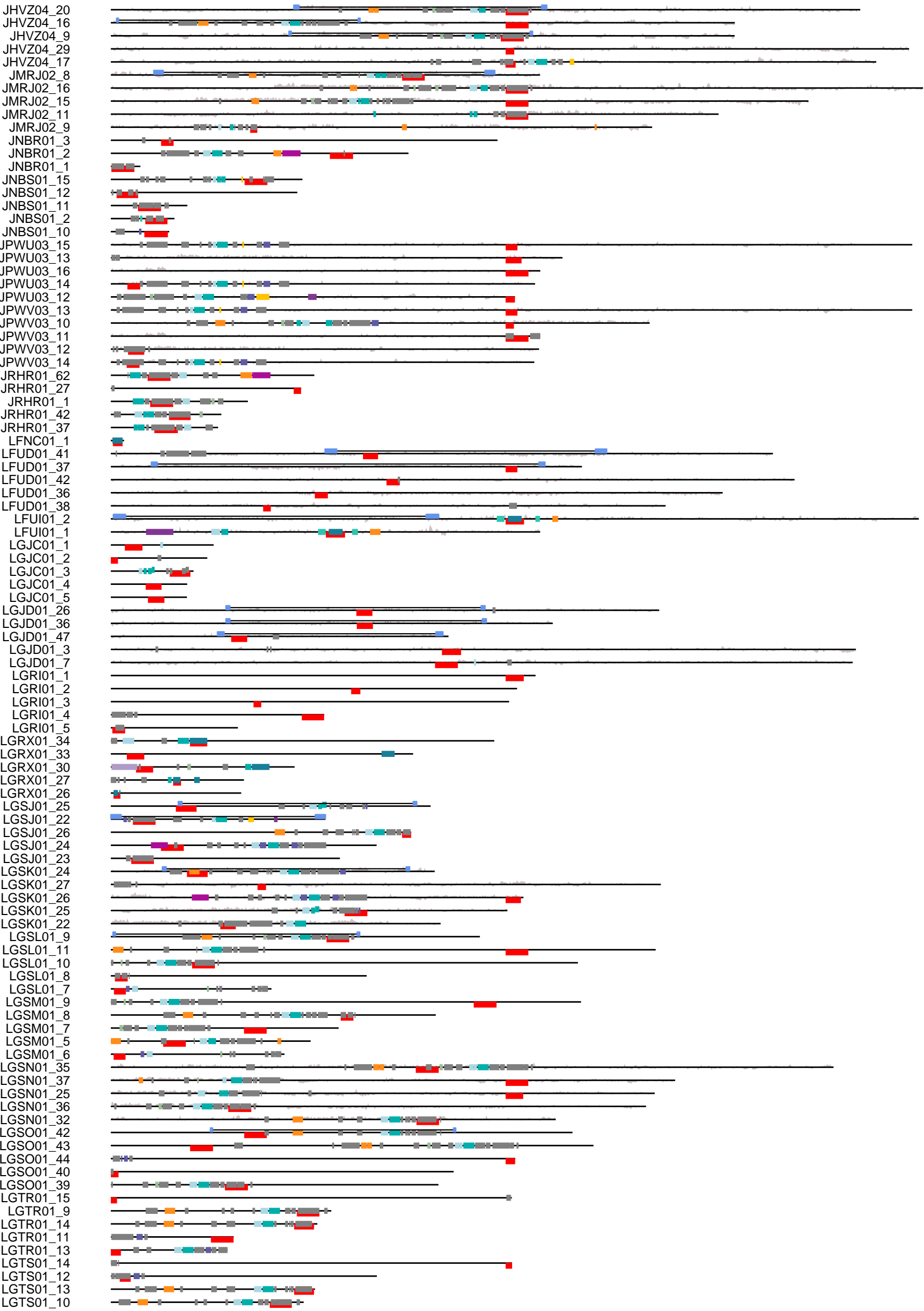

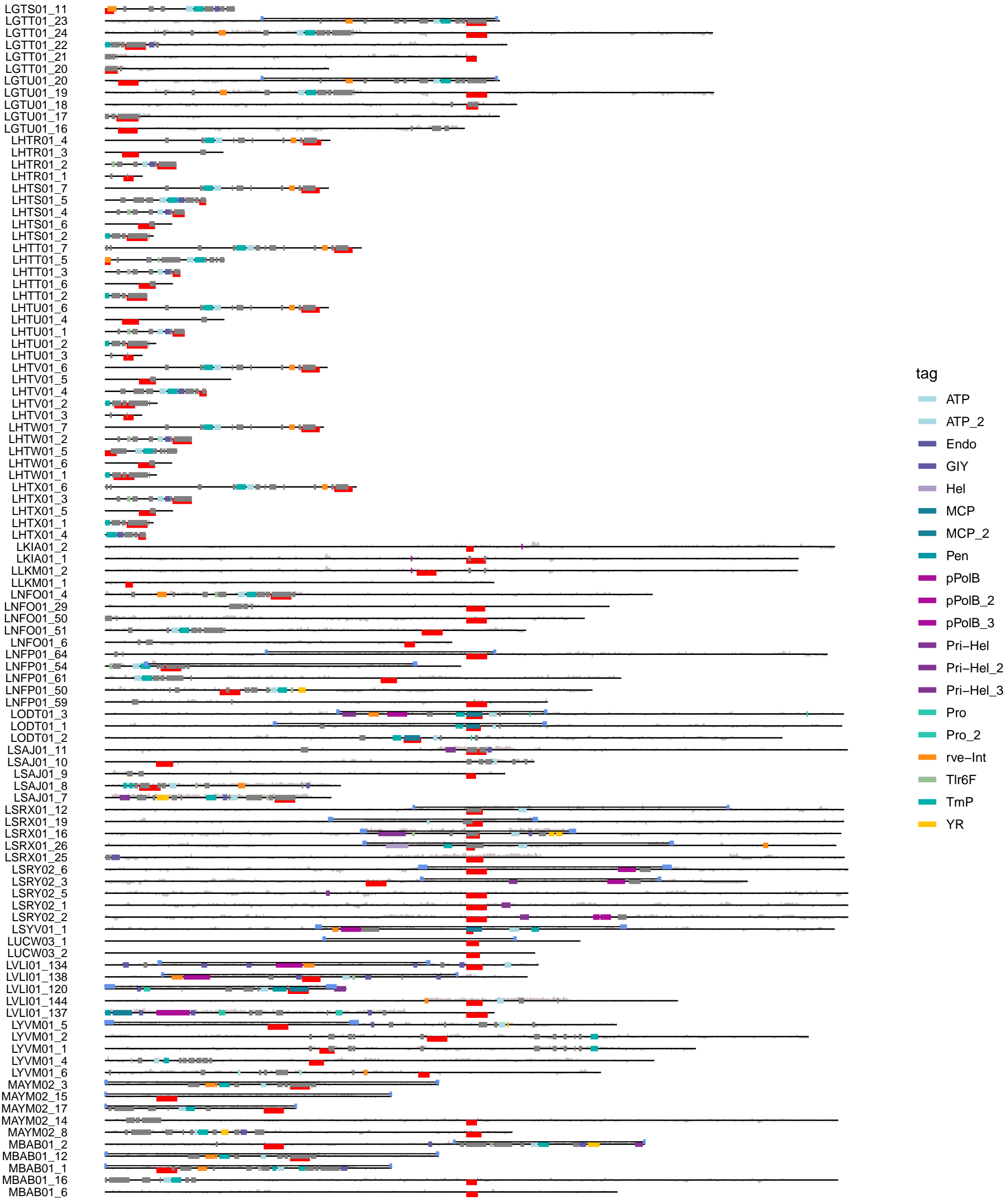

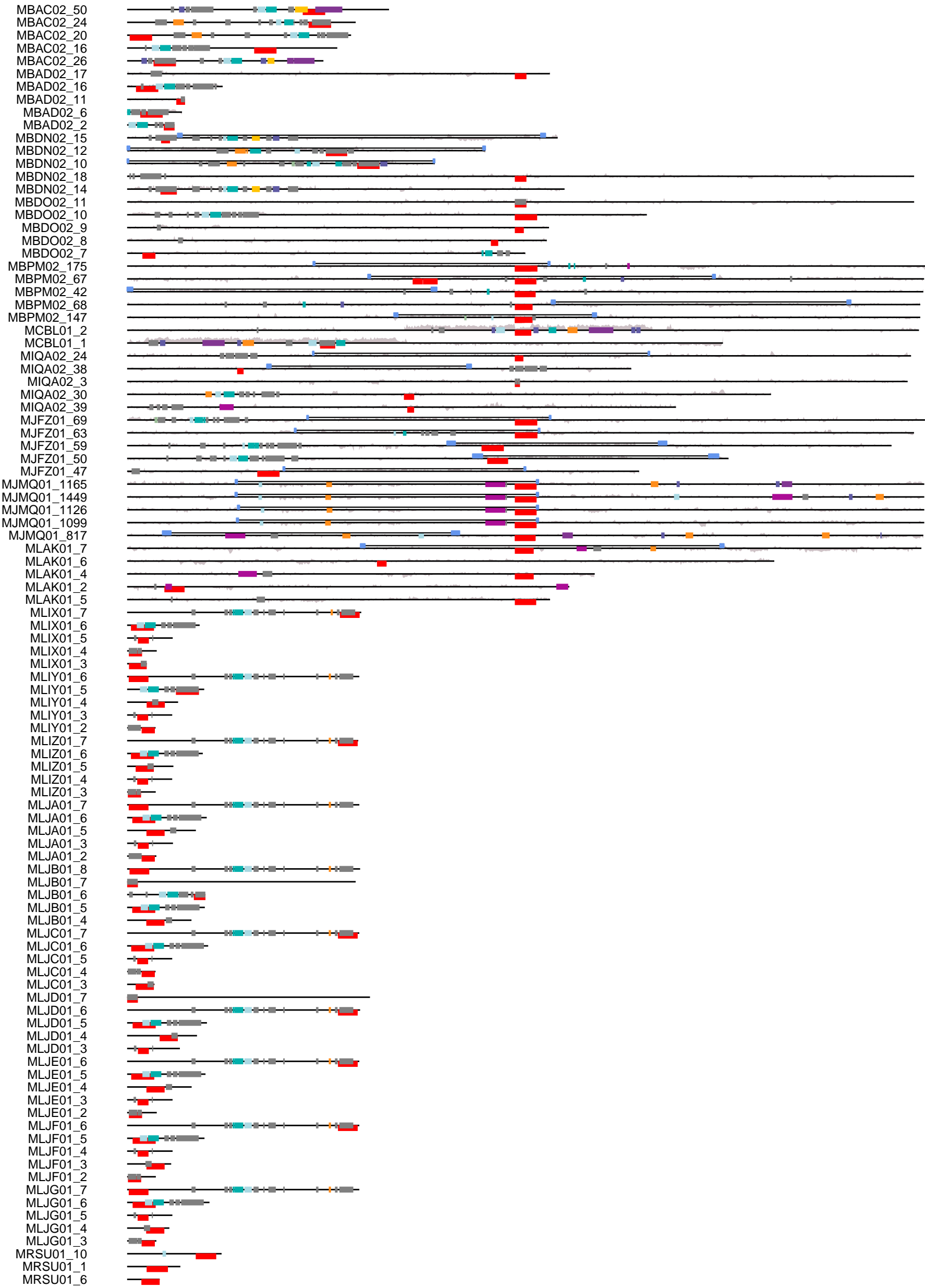

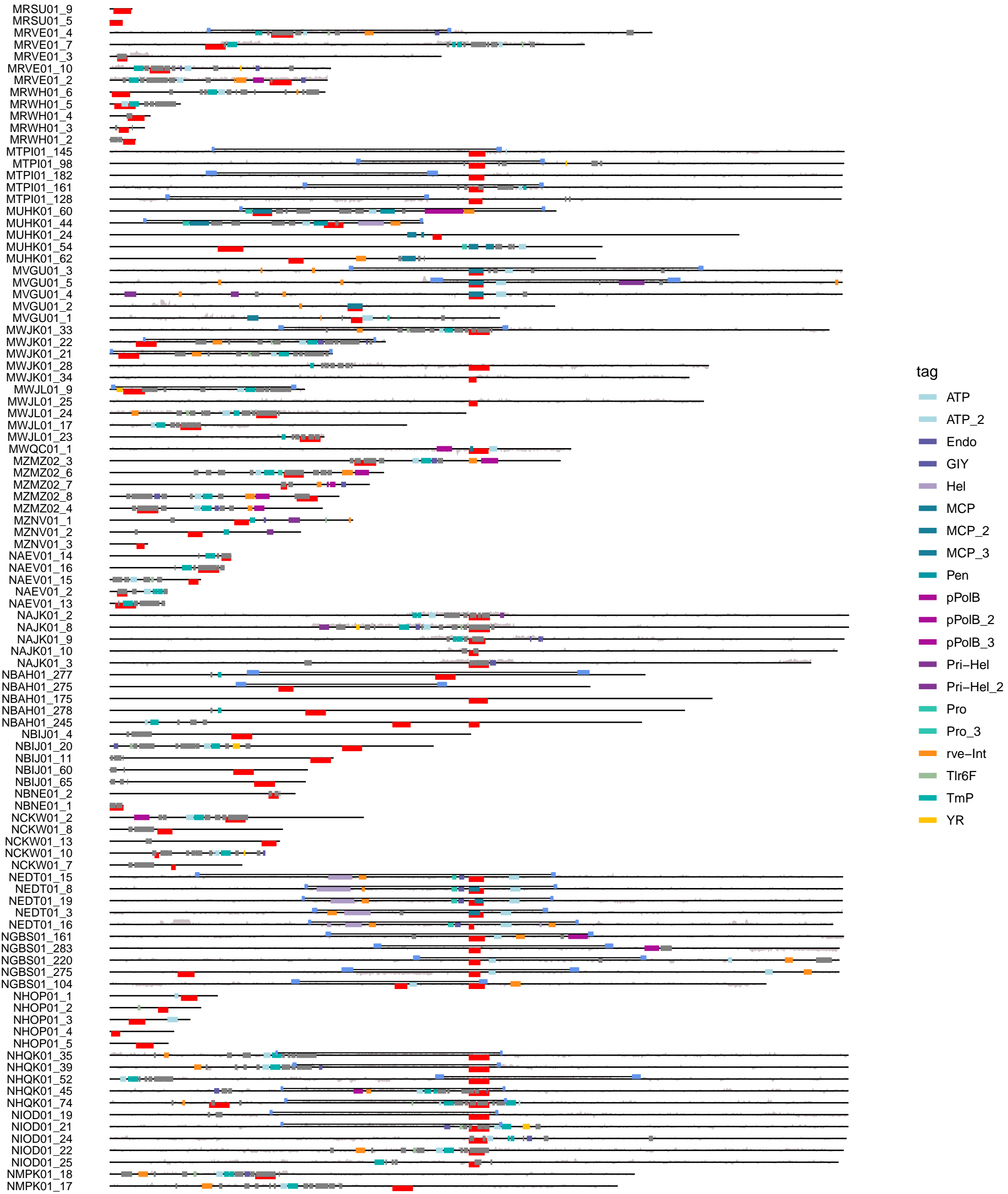

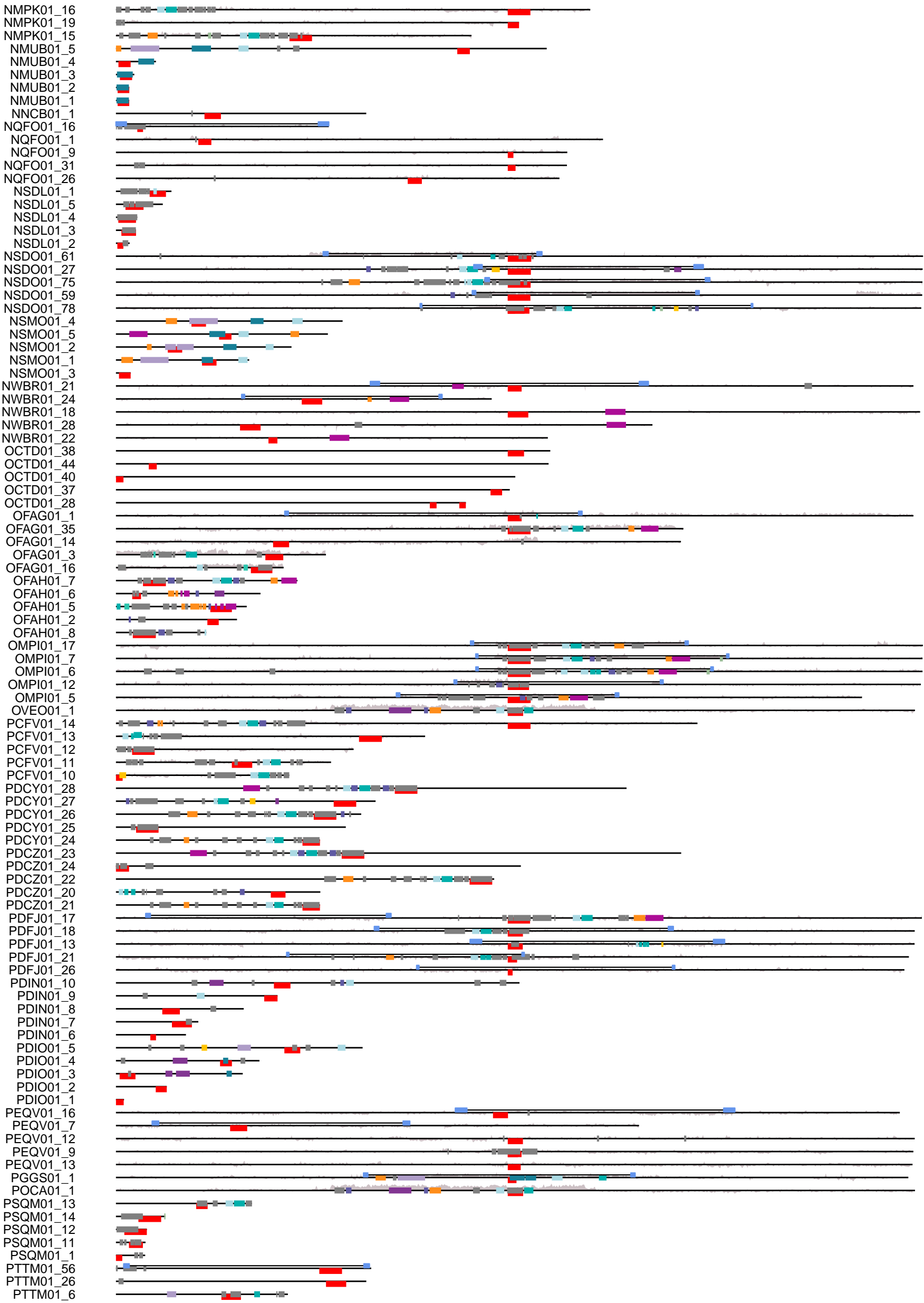

tag

- ATP
- ATP\_2
- Endo
- GIY
- Hel
- Hel\_3
- MCP\_2
- MCP\_3
- pPolB
- pPolB\_2
- pPolB\_3
- Pri-Hel\_2
- Pri-Hel\_3
- Pro
- rve-Int
- Tlr6F
- TmP
- YR

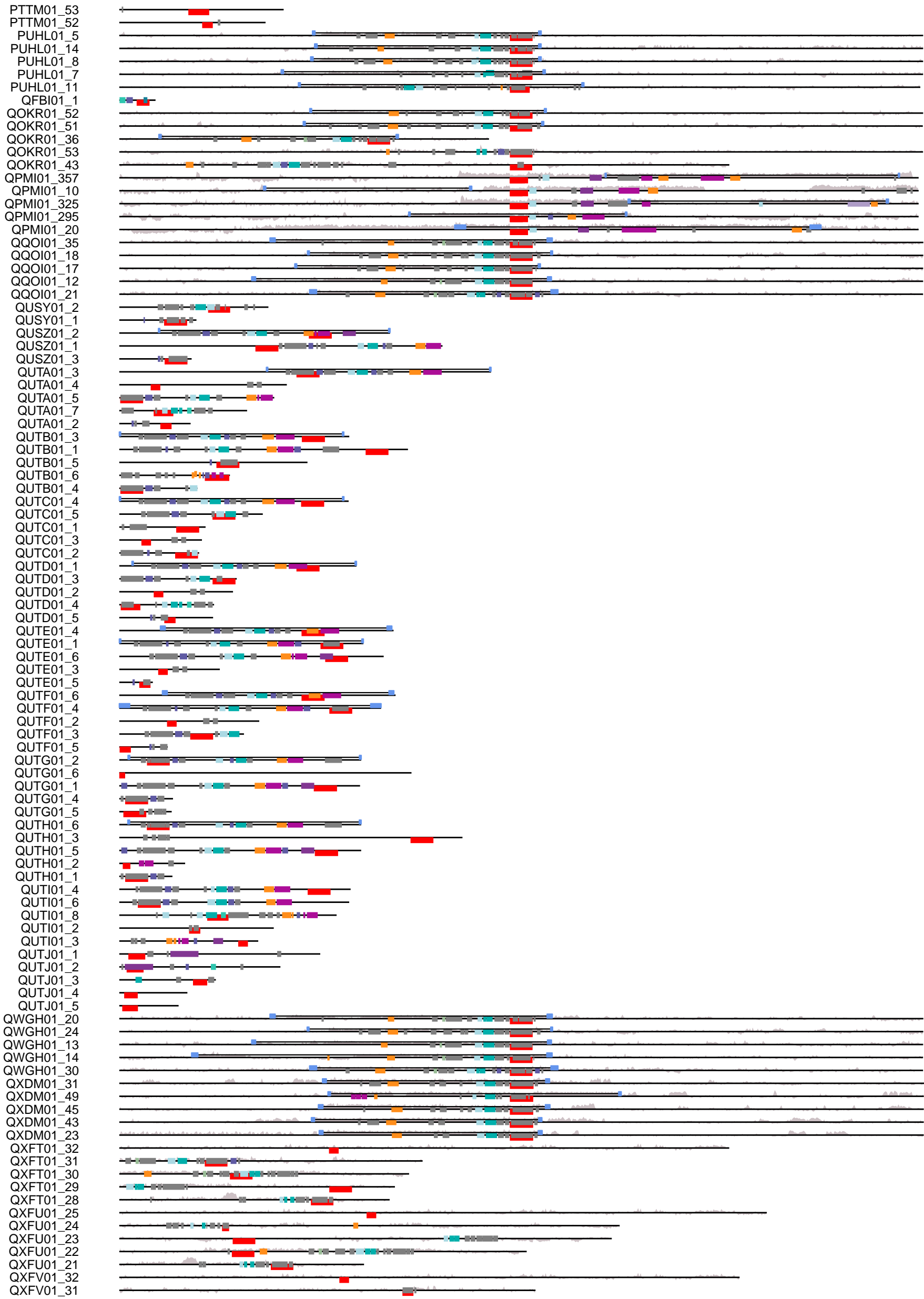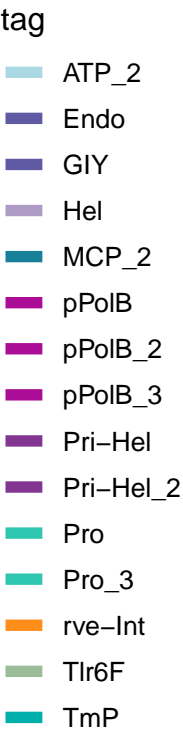

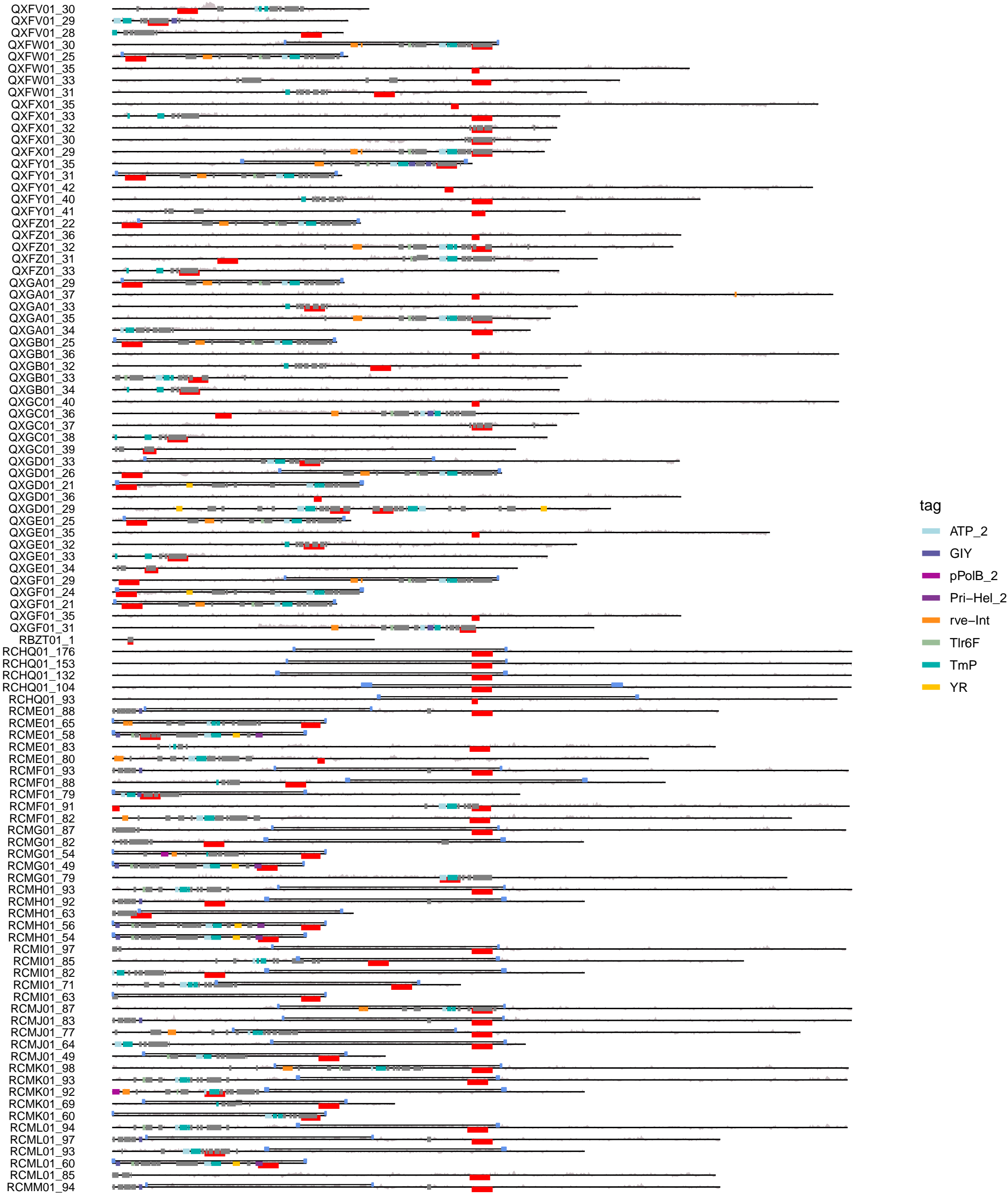

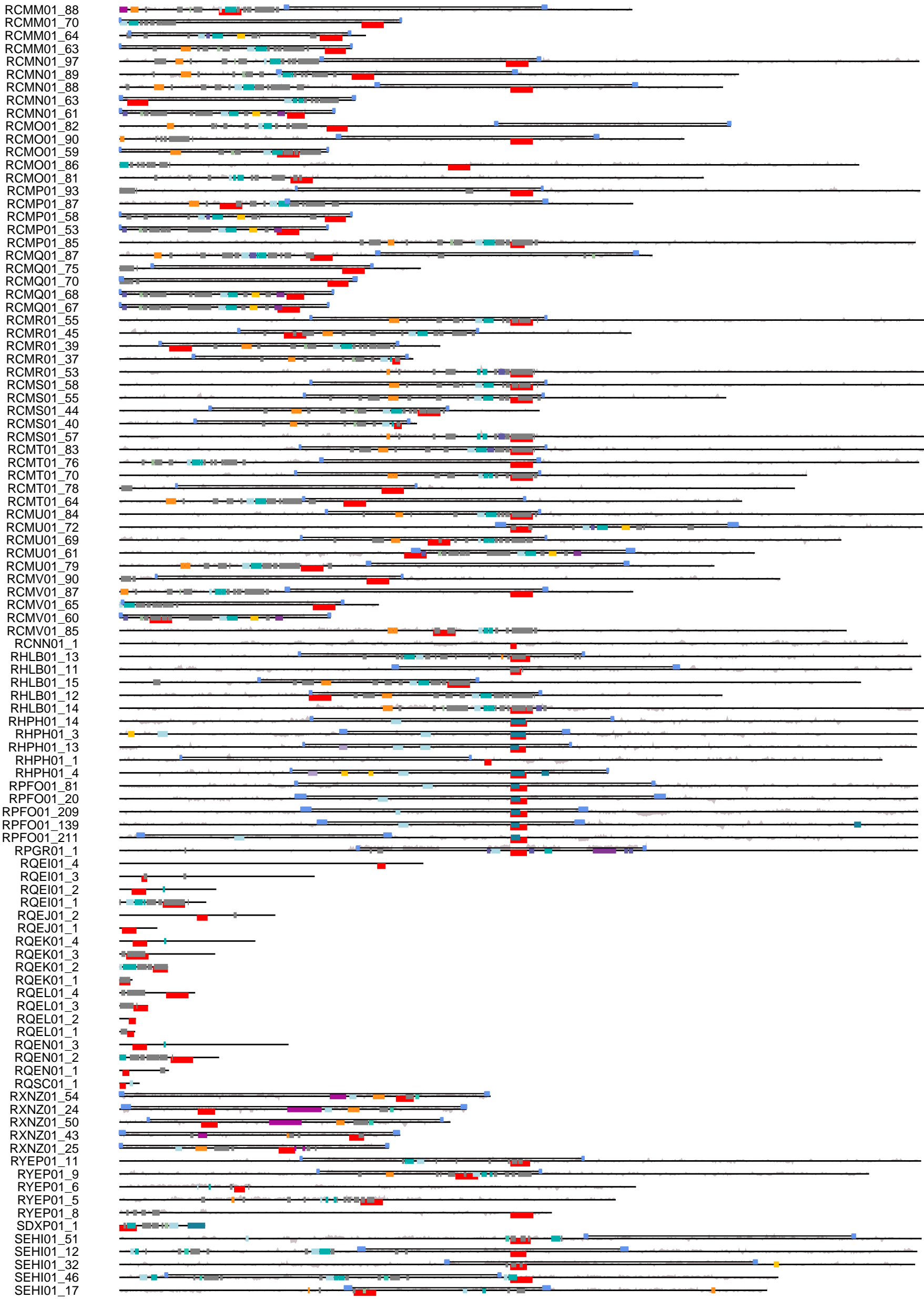

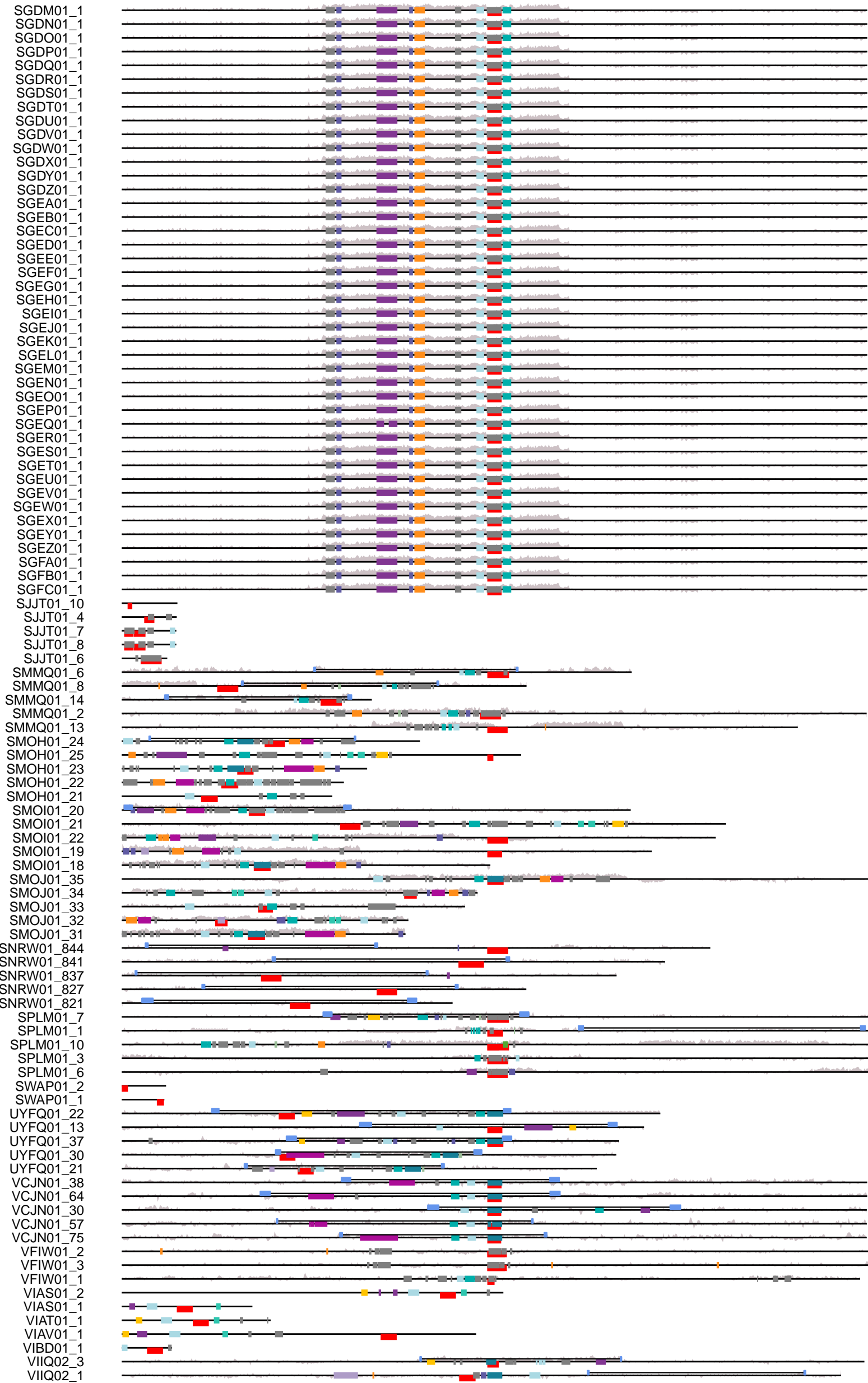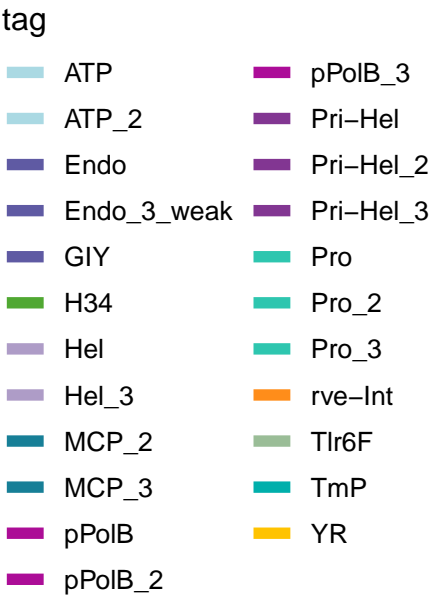

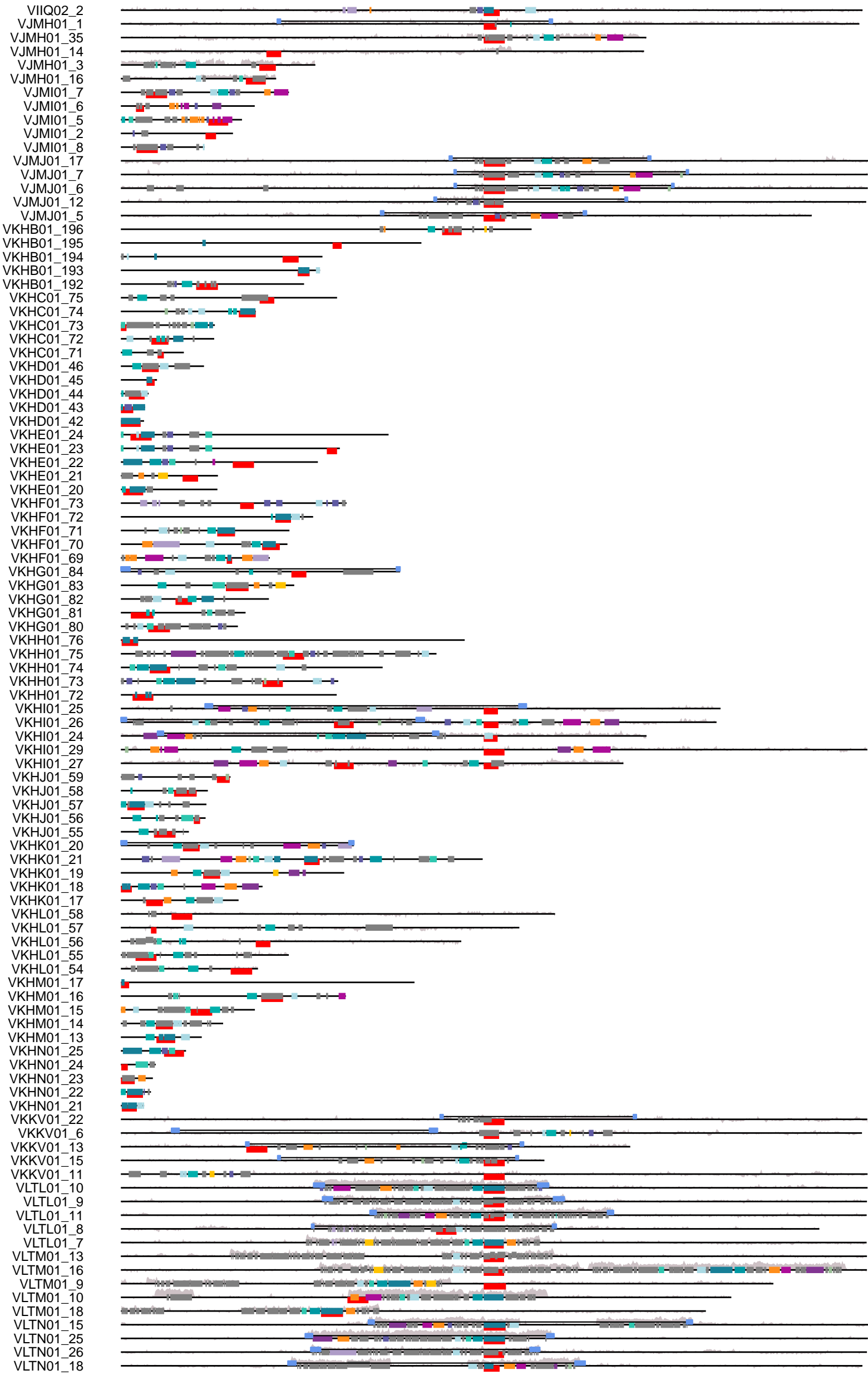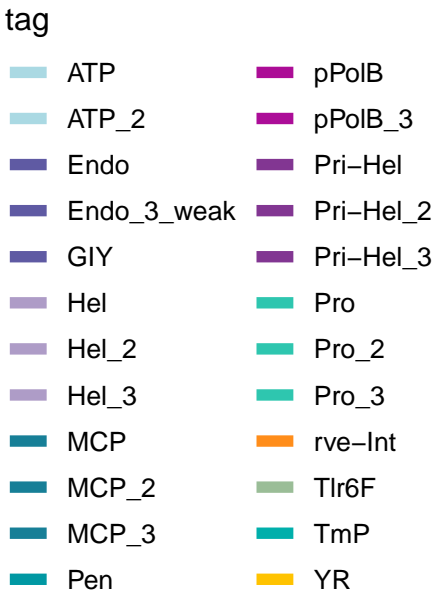

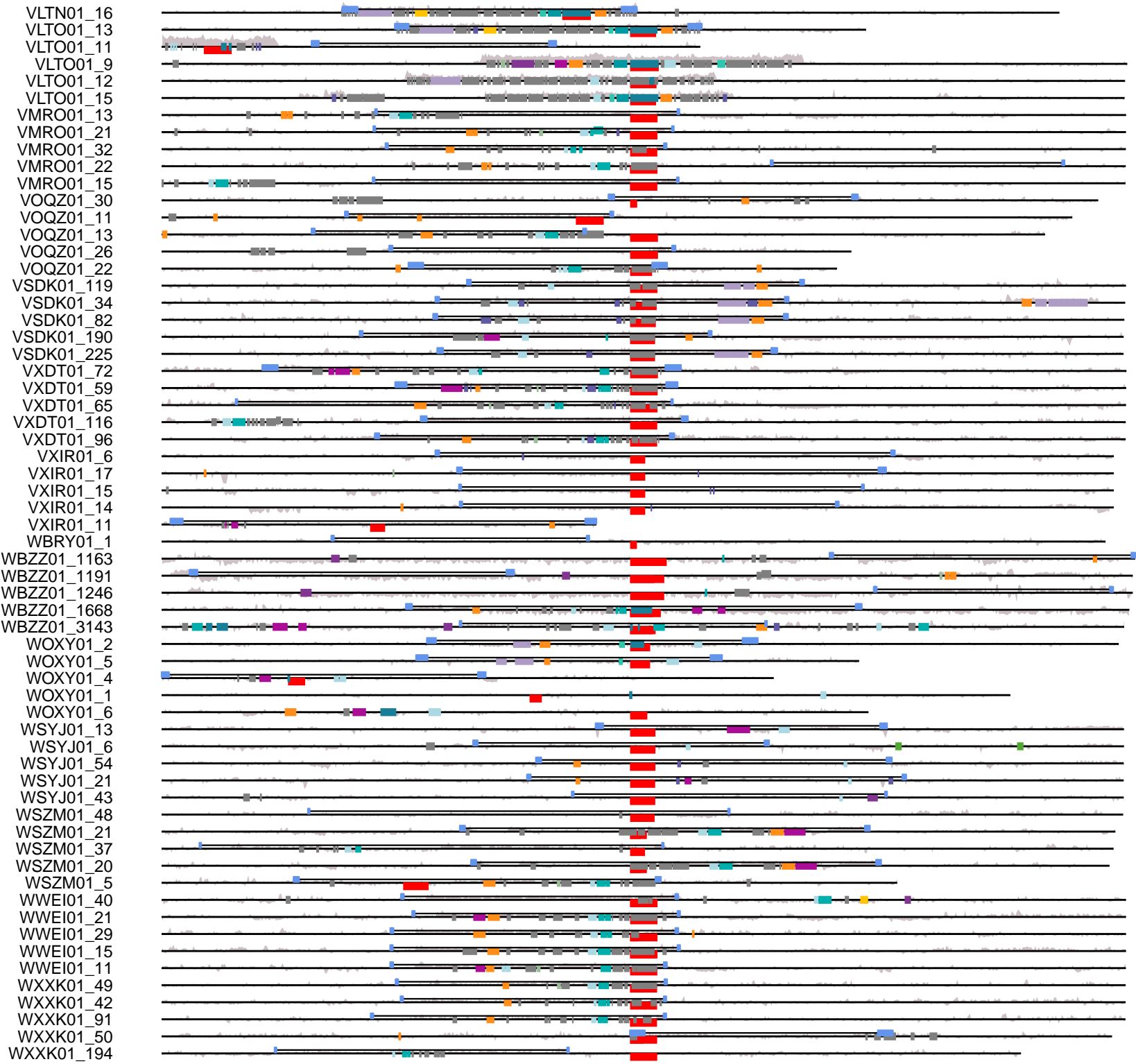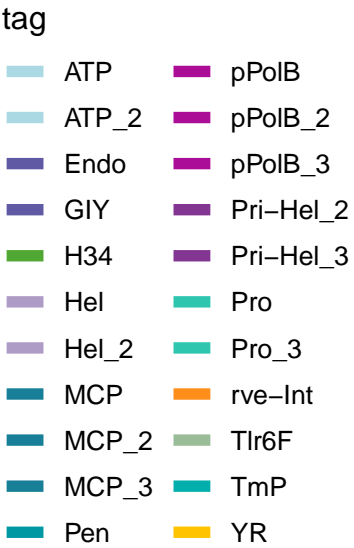

Supplement: Supplementary file 3 — Dataset S01 (PDF) [file pnas.2300465120.sd01.pdf]
